# Cilia–to–basement membrane signaling is a biomechanical driver in models of autosomal dominant polycystic kidney disease

**DOI:** 10.1172/JCI196814

**Published:** 2026-06-30

**Authors:** Manal Mazloum, Brice Lapin, Rushdi Alghamdi, Jessica Vandensteen, Martine Burtin, Pascal Houillier, Lydie Cheval, Gilles Crambert, Vicky Scata, Camille Cohen, Christoph Schell, Michael Rehman, Amandine Aka, Karim Ourahmoun, Rui Benedito, E. Wolfgang Kuehn, Stéphanie Descroix, Tilman Busch, Michael Köttgen, Serge Garbay, Marie-Christine Verpont, Ellie Tang, Brigitte Lelongt, Nicolas Cagnard, Stefan Somlo, Sylvie Coscoy, Fabiola Terzi, Amandine Viau, Frank Bienaimé

**Affiliations:** 1 Université Paris Cité, INSERM U1151, CNRS UMR 8235, Institut Necker Enfants Malades, Département Croissance et Signalisation, Paris, France.; 2 Assistance Publique-Hôpitaux de Paris, Hôpital Necker Enfants Malades, Service de Physiologie, Paris, France.; 3 Institut Curie, Université PSL, Sorbonne Université, CNRS UMR 168, Physics of Cells and Cancer, Paris, France.; 4 Department of Clinical Physiology, Faculty of Medicine, King Abdulaziz University, Jeddah, Saudi Arabia.; 5 Sorbonne Université et Université Paris Cité, Centre de Recherche des Cordeliers, INSERM, CNRS, Paris, France.; 6 Assistance Publique-Hôpitaux de Paris, Hôpital Européen Georges Pompidou, Service de Physiologie, Paris, France.; 7 Université Paris Cité, Centre de Recherche sur l’Inflammation, INSERM U1149, EMR 8252, Paris, France.; 8 Service de Néphrologie, Hôpital Bichat, Assistance Publique Hôpitaux de Paris, Paris, France.; 9 Institute of Surgical Pathology, Faculty of Medicine, Medical Center, University of Freiburg, Freiburg, Germany.; 10 Department of Internal Medicine, Yale School of Medicine, New Haven, Connecticut, USA.; 11 Institut du Cerveau et de la Moelle Épinière, INSERM U975, CNRS UMR 7225, Hôpital Pitié-Salpêtrière, Sorbonne Université, Paris, France.; 12 Molecular Genetics of Angiogenesis Group, Centro Nacional de Investigaciones Cardiovasculares, Madrid, Spain.; 13 Renal Division, Faculty of Medicine, Department of Medicine, Medical Center, University of Freiburg, Freiburg, Germany.; 14 Centre for Integrative Biological Signaling Studies, Freiburg, Germany.; 15 Sorbonne Université, CoRaKiD, INSERM UMRS 1155, Hôpital Tenon, Paris, France.; 16 Bioinformatic Platform, INSERM UMR 1163, Imagine Institute, Université Paris Cité, Paris, France.; 17 Department of Genetics, Yale School of Medicine, New Haven, Connecticut, USA.; 18 Université Paris Cité, Imagine Institute, Laboratory of Hereditary Kidney Diseases, INSERM UMR 1163, Paris, France.

**Keywords:** Genetics, Nephrology, Chronic kidney disease, Mouse models, Proteoglycans

## Abstract

Autosomal dominant polycystic kidney disease (ADPKD), the leading genetic cause of kidney failure, results from loss-of-function mutations in *PKD1*, encoding polycystin-1 (PC1). PC1 localizes to the primary cilium. In the absence of PC1, adverse signaling from the primary cilium orchestrates cyst formation, but the biomechanical underpinnings of this cilia-dependent cyst activation (CDCA) remain unclear. Combining tubule-specific orthologous mouse models with a tubule-on-chip platform, we show that PC1 and cilia govern the composition, mechanical properties, and shape of the tubular basement membrane (TBM), the principal rigid determinant of tubule geometry. PC1 loss triggered TBM thinning, heparan sulfate enrichment, and deformation, leading to distension, preferentially of the distal nephron. These changes were driven by a cilia-dependent transcriptional program, with GLIS2 — a key CDCA effector — participating as a downstream mediator. Reduction of TBM stiffness amplified *Pkd1*^–/–^ tubule-on-chip dilation and increased cyst formation in vivo. Conversely, increasing luminal pressure through ureteral obstruction induced disproportionate distension of *Pkd1*-deficient tubules and triggered an irreversible cystogenic program. Together, these findings establish a TBM-centered biomechanical model of ADPKD in which tubule deformation is governed by both basolateral and luminal mechanical factors and identify the cilium/TBM axis, operating in part through GLIS2, as a central driver of cystogenesis.

## Abstract 2

This work unveils basement membrane remodeling and altered tubule mechanics as critical drivers of cyst formation in autosomal dominant kidney disease. It further pinpoints candidate genes that may be responsible for these phenomena. Overall, our results suggest that targeting basement membrane remodeling is a potential therapeutic option in autosomal dominant polycystic kidney disease.

## Graphical abstract

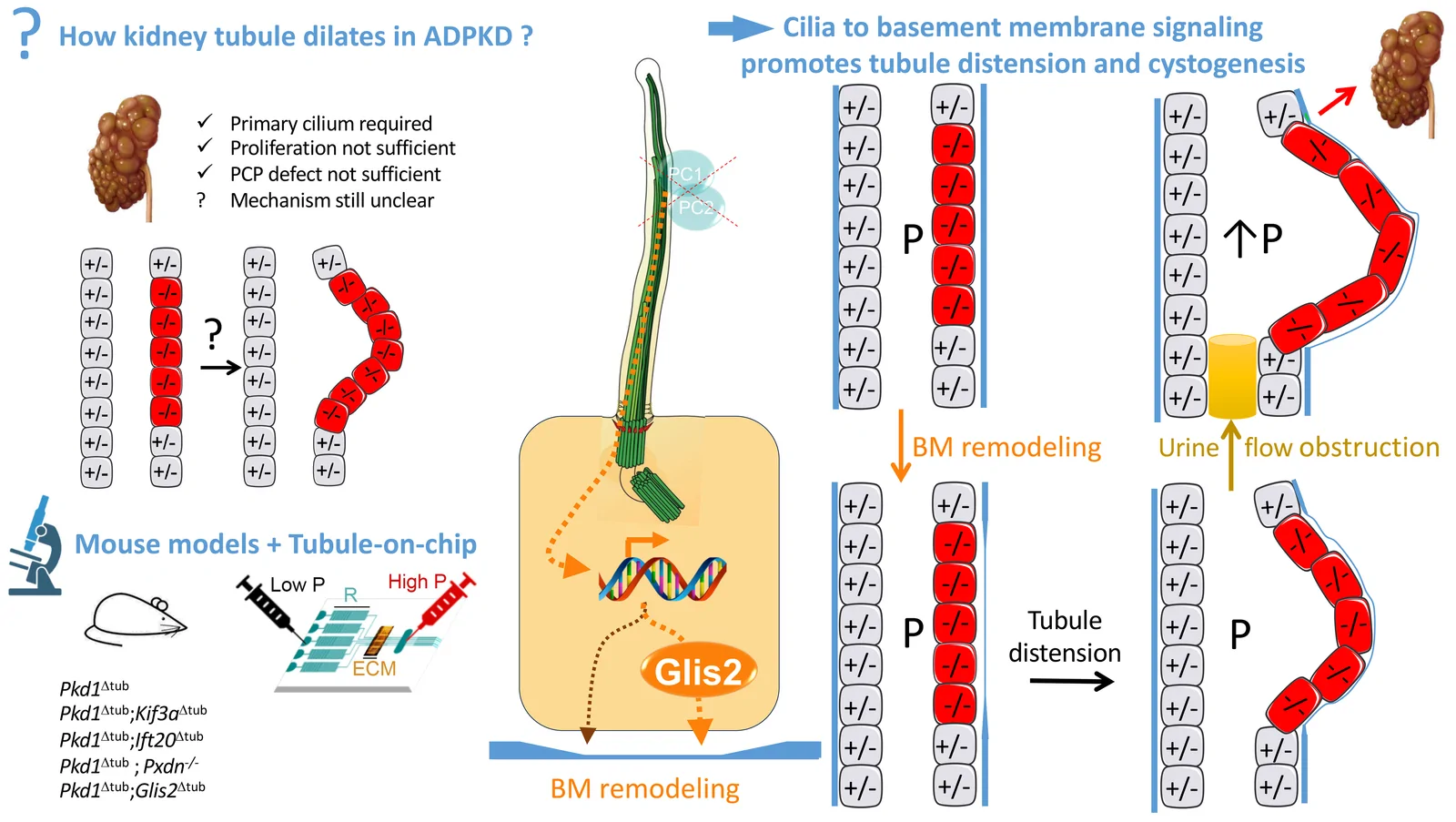

## Introduction

Autosomal dominant polycystic kidney disease (ADPKD) is the most common inherited renal disorder, accounting for 5%–10% of kidney failure cases. It is characterized by the progressive transformation of a subset of renal tubules into cysts that grow in number and size, increasing renal mass up to 20-fold and progressively impairing kidney function, with most patients reaching kidney failure by the sixth decade (1). Only modest benefit is gained from blood pressure control and the vasopressin V2R antagonist tolvaptan, the sole approved treatment for ADPKD (2).

ADPKD is caused by mutations inactivating the complex formed by polycystin-1 (PC1; encoded by *PKD1*), a large orphan receptor, and PC2 (encoded by *PKD2*), a cation channel. This complex localizes mainly to primary cilia, filiform organelles protruding from the apical surface of tubular cells that integrate chemical and mechanical cues. Although patients carry heterozygous germline mutations, cystogenesis is triggered only when functional PC1/PC2 falls below a critical threshold, most often through somatic inactivation of the remaining allele (3). Yet, how cysts form remains unresolved.

Cell proliferation sustains cyst growth downstream of polycystin inactivation but is insufficient on its own to initiate it: tubular proliferation also rises after nephron reduction, ischemia-reperfusion injury, or albuminuria without cyst formation (4–6). Planar cell polarity defects have been proposed to cause cysts through loss of oriented cell division or convergent extension (7, 8), but recent evidence indicates that planar cell polarity defects are not sufficient to initiate cyst growth (9, 10). Likewise, several pathways promote cyst growth (mTOR, cAMP, metabolic rewiring), yet none appears active during early tubule dilation (11). The primary morphogenetic events causing tubule dilation thus remain only partially understood.

The primary cilium is instrumental in cystogenesis. Ciliary PC1/PC2 is required to prevent cysts (12), and genetic cilia ablation prevents cyst formation in *Pkd1* mutant mice (11, 13), supporting a cilia-dependent cyst activation (CDCA) pathway. GLIS2 was recently identified as a critical transcriptional effector of CDCA, though how it induces tubule dilation is unclear (14). Two other cilia-regulated genes promote cystogenesis — *Ccl2* (13) and *Cdk1* (15), acting in macrophage recruitment and tubular proliferation — but, unlike cilia ablation, their loss slows cyst expansion rather than preventing early dilation of *Pkd1*-deficient tubules (13, 15), implying additional ciliary functions in cystogenesis.

In higher metazoans, the ECM is a key determinant of tissue geometry and mechanics. In the kidney, decellularization shows that the ECM — largely composed of juxtaposed tubular basement membranes (TBMs) — retains nephron shape and the tubule compliance that accommodates the luminal pressure required for kidney function (16). The TBM comprises interacting collagen IV and laminin networks linked by heparan sulfate (HS) proteoglycans and nidogen (17). Through their hydrophilic carbohydrate chains, HS proteoglycans promote basement membrane (BM) hydration and deformability (18), whereas the collagen and laminin networks confer rigidity, further increased by covalent collagen IV cross-links (19, 20). BM biomechanics are thus largely set by thickness, composition, and cross-linking (21).

The role of TBM mechanics in ADPKD has not been systematically investigated. Because intratubular pressure is not raised in cysts of rodent models, early researchers proposed that cysts arise from increased TBM deformability (22); however, early studies — mostly using nonorthologous models — failed to show consistent changes in TBM mechanics, structure, or composition (22, 23).

Here, we combined tubule-specific *Pkd1*-deletion mouse models, with or without concomitant cilia ablation, and a tubule-on-chip model with tunable ECM properties to dissect the interplay among cilia, *Pkd1*, TBM, and tubular dilation.

## Results

### Pkd1 loss triggers cilia-dependent proximal and distal nephron dilation through distinct mechanisms.

**Figure 1:**
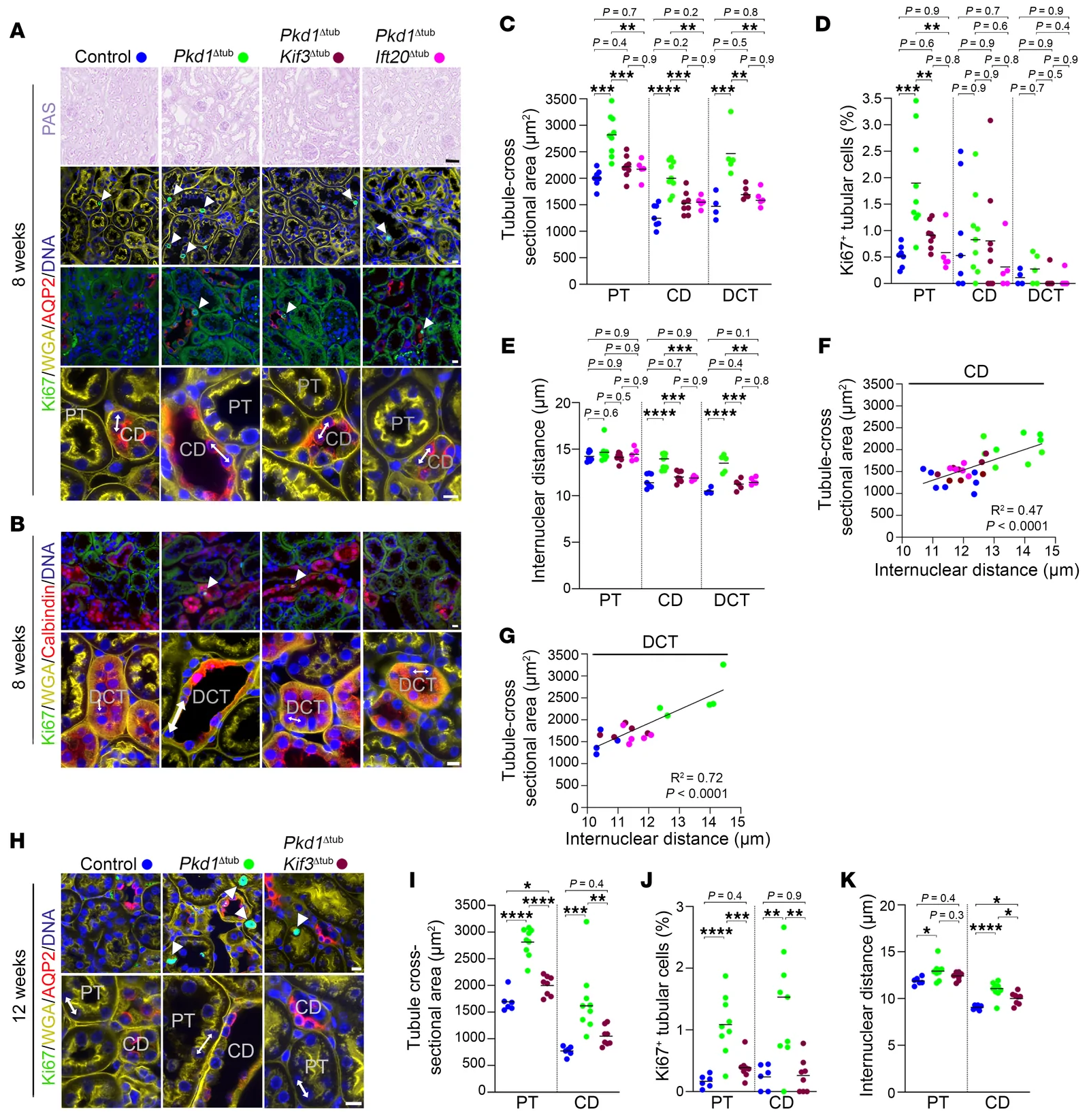
*Pkd1* deletion drives cilia-dependent distal tubule distension independently of cell proliferation. (**A** and **B**) Periodic acid Schiff (PAS) staining and labeling of Ki67 (which stains proliferating cells), DNA, wheat germ agglutinin (WGA; which stains the brush border of PTs and all BMs), and aquaporin 2 (AQP2; a CD marker) (**A**) or calbindin (a marker of DCTs) (**B**) of kidneys from 8-week-old control, *Pkd1*^Δtub^, *Pkd1*^Δtub^ *Kif3a*^Δtub^, and *Pkd1*^Δtub^ *Ift20*^Δtub^ male mice. Arrows: examples of internuclear distance measurements. Scale bars: 10 μm. (**C**–**E**) Quantification of mean PT, CD, and DCT cross-sectional area (**C**); proliferation index (percentage of Ki67^+^ cells; **D**); and internuclear distance (**E**) in PTs, CDs, and DCTs of kidneys from 8-week-old control, *Pkd1*^Δtub^, *Pkd1*^Δtub^ *Kif3a*^Δtub^, and *Pkd1*^Δtub^ *Ift20*^Δtub^ mice. (**F** and **G**) Linear regression of tubule cross-sectional area and internuclear distance for CDs (**F**) and DCTs (**G**) in the same mice groups at 8 weeks. (**H**) Labeling of Ki67, DNA, WGA, and AQP2 of kidneys from 12-week-old control, *Pkd1*^Δtub^, and *Pkd1*^Δtub^ *Kif3a*^Δtub^ mice. Arrows: examples of internuclear distance measurements. Scale bars: 10 μm. (**I**–**K**) Quantification of mean tubule cross-sectional area (**I**), proliferation index (Ki67^+^ cells) (**J**), and internuclear distance (**K**) in PTs and CDs of kidneys from 12-week-old control, *Pkd1*^Δtub^, and *Pkd1*^Δtub^ *Kif3a*^Δtub^ mice. Each dot represents 1 male mouse. One-way ANOVA followed by Tukey-Kramer test: *P < 0.05, **P < 0.01, ***P < 0.001, ****P < 0.0001, or the indicated *P* value. *Pkd1^fl/fl^* and *Pkd1^fl/fl^* *Ift20^fl/fl^* littermates lacking cre or rtTA transgene were used as control. Confirmatory analyses on distal collecting tubules were only performed in the first cohort of mice analyzed.

To gain insights into the early morphogenic events underlying tubule dilation, we first studied male mice with a postdevelopmental inactivation of *Pkd1* (*Pkd1*^Δtub^) with or without simultaneous ablation of primary cilium (*Kif3a* or *Ift20* inactivation: *Pkd1*^Δtub^ *Kif3a*^Δtub^ and *Pkd1*^Δtub^ *Ift20*^Δtub^) at a very early stage of the disease, that is, 2 weeks after the completion of doxycycline treatment (i.e., 8 weeks of age), when tubules display only slight dilations (Figure 1A). We used the Pax8rtTA system, which drives functional Cre expression along the nephron (Supplemental Figure 1A; supplemental material available online with this article; https://doi.org/10.1172/JCI196814DS1), efficiently excises floxed *Pkd1* alleles (Supplemental Figure 1B), and achieves near complete cilia loss in *Pkd1*^Δtub^ *Kif3a*^Δtub^ tubules (Supplemental Figure 1C). Morphometric analysis confirmed that *Pkd1* loss induces an increase in cross-sectional areas of proximal tubules (PTs) and distal tubules (consisting of collecting ducts [CDs] and distal convoluted tubules [DCTs]), which was prevented by concomitant cilia disruption following *Kif3a* or *Ift20* inactivation (Figure 1, A–C). Ki67 labeling revealed that PC1 deficiency increased cell proliferation in PTs (Figure 1, A and D), but not in the distal nephron (Figure 1, A, B, and D). Instead, we observed increased tubular cell stretching in CDs and DCTs, as reflected by an increase in the mean distance between 2 adjacent nuclei (later referred to as internuclear distance; Figure 1, A, B, and E). Linear regression analysis showed that distension (i.e., increase in internuclear distance) was the main factor explaining distal nephron dilation at this stage (Figure 1, F and G). At a later time point, distension and increased proliferation were observed in both proximal and distal *Pkd1*^–/–^ tubules (Figure 1, H–K). Genetic cilia ablation (e.g., *Kif3a* or *Ift20* inactivation) rescued PC1-deficient distal nephron distension, tubular cell proliferation, and cyst development (Figure 1, H–K). Analysis of 18-week-old animals confirmed that cilia ablation fully rescued tubule deformation in *Pkd1*^Δtub^ mice (Supplemental Figure 2). Collectively, these results identify cilia-driven tubule distension as an important factor responsible for the early dilation of PC1-deficient tubules.

### Preferential distal tubule distension correlates with specific changes of the TBM.

**Figure 2:**
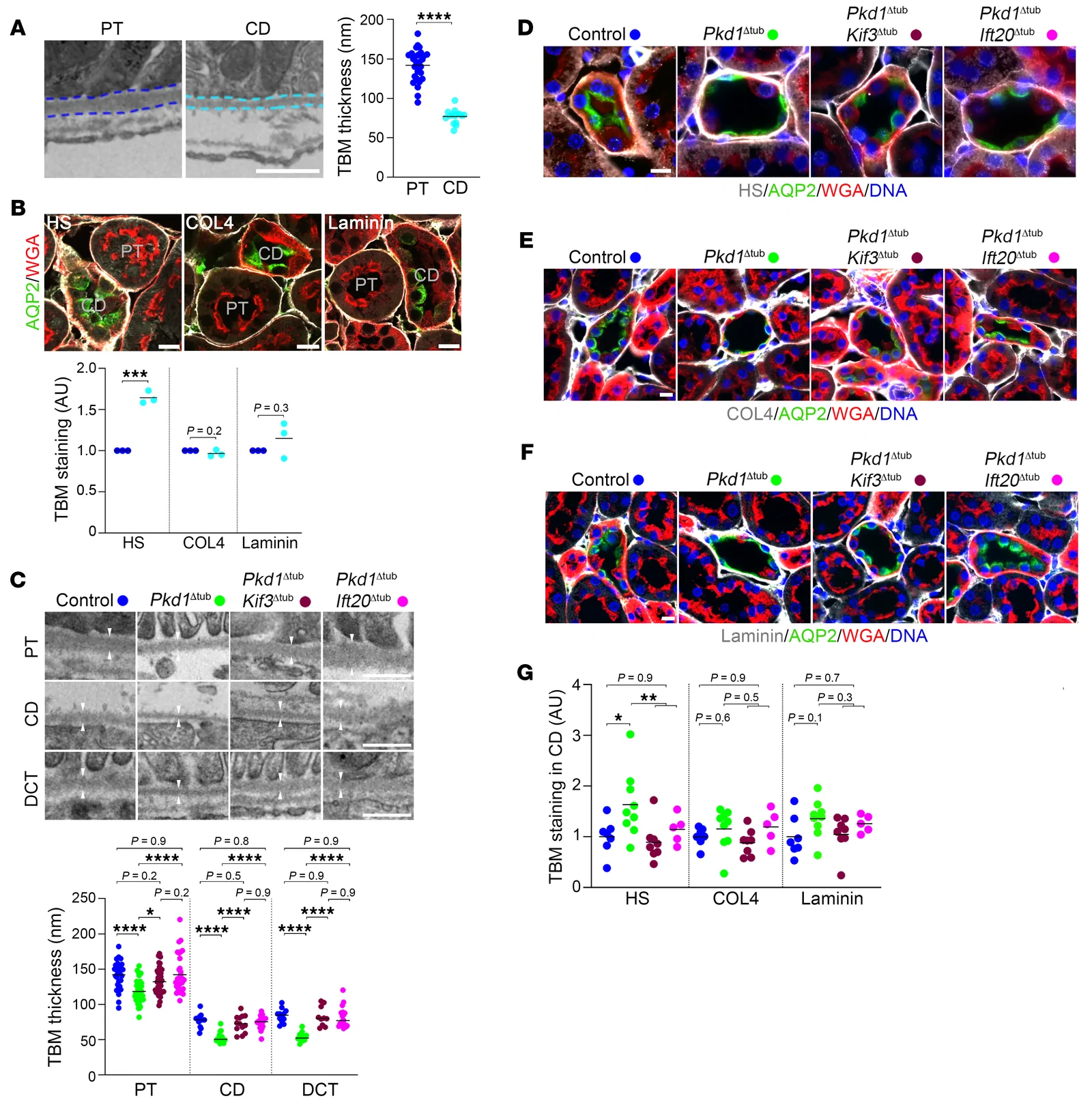
*Pkd1* deletion induces cilia-dependent BM remodeling. (**A**) Transmission electron microscopy and quantification of PT and CD TBM thickness in 8-week-old control mice. Dashed lines underline TBM. Each dot represents 1 tubule (mean of 3–21 measurements per tubule; *n* = 3 mice). Scale bar: 0.5 μm. Student’s *t* test: ****P < 0.0001. (**B**) Labeling of PT brush border (WGA), CD (AQP2), and HS (left panel); collagen IV (COL4; middle panel); or laminin (right panel) and quantification of HS, COL4, and laminin signal intensity in 8-week-old control mice. Each dot represents 1 male mouse. Scale bars: 10 μm. Student’s *t* test: ***P < 0.001. (**C**) Transmission electron microscopy and quantification of TBM thickness of PTs, CDs, and DCTs from 8-week-old control, *Pkd1*^Δtub^, *Pkd1*^Δtub^ *Kif3a*^Δtub^, and *Pkd1*^Δtub^ *Ift20*^Δtub^ mice (i.e., 2 weeks after the end of doxycycline treatment). Each dot represents 1 tubule (mean of 8–20 measurements per tubule; *n* = 4–5 mice per genotype). Scale bars: 0.5 μm. One-way ANOVA followed by Tukey-Kramer test: *P < 0.05, ****P < 0.0001, or the indicated *P* value. (**D**–**G**) Staining and quantification of HS (**D** and **G**), COL4 (**E** and **G**), and laminin (**F** and **G**) in CDs labeled with AQP2 from 8-week-old control, *Pkd1*^Δtub^, *Pkd1*^Δtub^ *Kif3a*^Δtub^, and *Pkd1*^Δtub^ *Ift20*^Δtub^ mice. Each dot represents 1 male mouse. Scale bars: 10 μm. One-way ANOVA followed by Tukey-Kramer test: *P < 0.05, **P < 0.01, or the indicated *P* value. Data from *Pkd1*^Δtub^ *Kif3a*^Δtub^ and *Pkd1*^Δtub^ *Ift20*^Δtub^ mice were pooled for this statistical analysis. *Pkd1^fl/fl^* and *Pkd1^fl/fl^* *Kif3a^fl/fl^* littermates lacking cre or rtTA transgene were used as control.

As tubule shape is determined by TBM geometry (16), we reasoned that CD distension could be linked to specific TBM properties. TBM stiffness depends on its thickness and composition: laminin and cross-linked collagen IV provide rigidity, while the sugar moieties of HS and chondroitin sulfate (CS) confer flexibility by increasing BM hydration (18). Studying these parameters in basal conditions, we noticed that CDs had thinner TBMs compared with PTs with higher HS content, suggesting an increased compliance (Figure 2, A and B), as historically suggested by pressure-volume measurements on isolated rabbit tubules (16). We further observed that distal nephron distension in *Pkd1*^Δtub^ mice was associated with prominent TBM thinning (Figure 2C). TBM thinning also occurred in PTs but to a lesser extent than in CDs or DCTs (Figure 2C). A careful inspection of multiple tubule sections via transmission electron microscopy failed to detect any perforation or tubular cell protrusion through the TBM (Figure 2C and Supplemental Figure 3). Cilia ablation abolished the ability of PC1-deficient tubular cells to induce TBM thinning (Figure 2C). We further observed a specific increase in HS immunoreactivity of the TBM of PC1-deficient tubules undergoing distension, which was prevented by cilia disruption, without notable changes in collagen IV and laminin labeling (Figure 2, D–G). These results indicate that *Pkd1* loss is associated with a cilia-dependent remodeling of the TBM. They also suggest that intrinsic properties of distal nephron TBMs may render this segment permissive to distension. Detailed transmission electron micrographs of TBMs from male mice are presented in Supplemental Figure 3.

We further examined tubular remodeling in kidneys from *Pkd1*^Δtub^ female mice, which display slower disease progression, at successive time points to better define the temporal relationship between TBM thinning and tubule distension (Supplemental Figure 4). TBM thickness was already decreased in *Pkd1*-deficient CDs at 8 weeks of age (Supplemental Figure 4, A–E), whereas an increase in internuclear distance and HS staining was only observed later at 12 weeks (Supplemental Figure 4, F–J). TBM thinning persisted at 18 weeks in *Pkd1*-deficient kidneys (Supplemental Figure 4, K–M), although multilayering of the TBM was occasionally observed (Supplemental Figure 4N). These results identify TBM thinning as an early event that precedes tubule distension and HS enrichment and that persists in overtly cystic kidneys.

### TBM remodeling and tubule distension are more prominent features of cystogenesis driven by Pkd1 loss than by cilia ablation.

**Figure 3:**
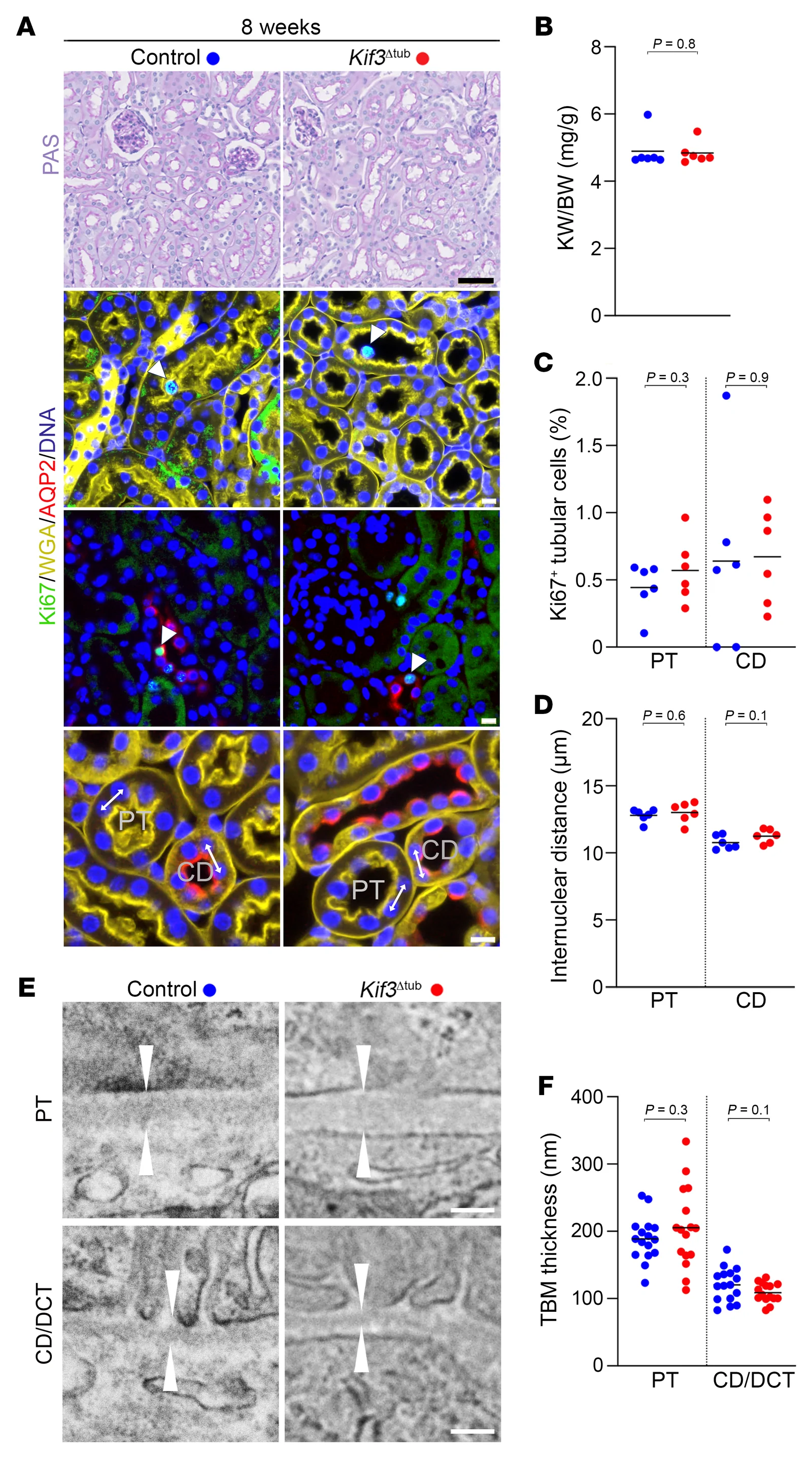
*Kif3a* inactivation alone does not lead to tubule distension. (**A**) PAS staining and labeling of Ki67 (which stains proliferating cells; arrowheads), DNA, WGA (which stains the brush border of PTs and all BMs), and AQP2 (a CD marker) of kidneys from 8-week-old control or *Kif3a*^Δtub^ mice. Arrows: examples of internuclear distance measurements. Scale bars: 10 μm. (**B**–**D**) Quantification of KW/BW (**B**), proliferation index (percentage of Ki67^+^ cells; **C**), and internuclear distance (**D**) in PTs and CDs of kidneys from 8-week-old control or *Kif3a*^Δtub^ mice. (**E** and **F**) Transmission electron microscopy (**E**) and quantification (**F**) of TBM thickness of PTs and CDs from 8-week-old control and *Kif3a*^Δtub^ littermate mice (i.e., 2 weeks after the end of doxycycline treatment). Each dot represents 1 tubule (mean of 8–20 measurements per tubule; *n* = 3 mice per genotype). Scale bars: 200 nm. *P* values are from 1-way ANOVA followed by Tukey-Kramer test.

**Figure 4:**
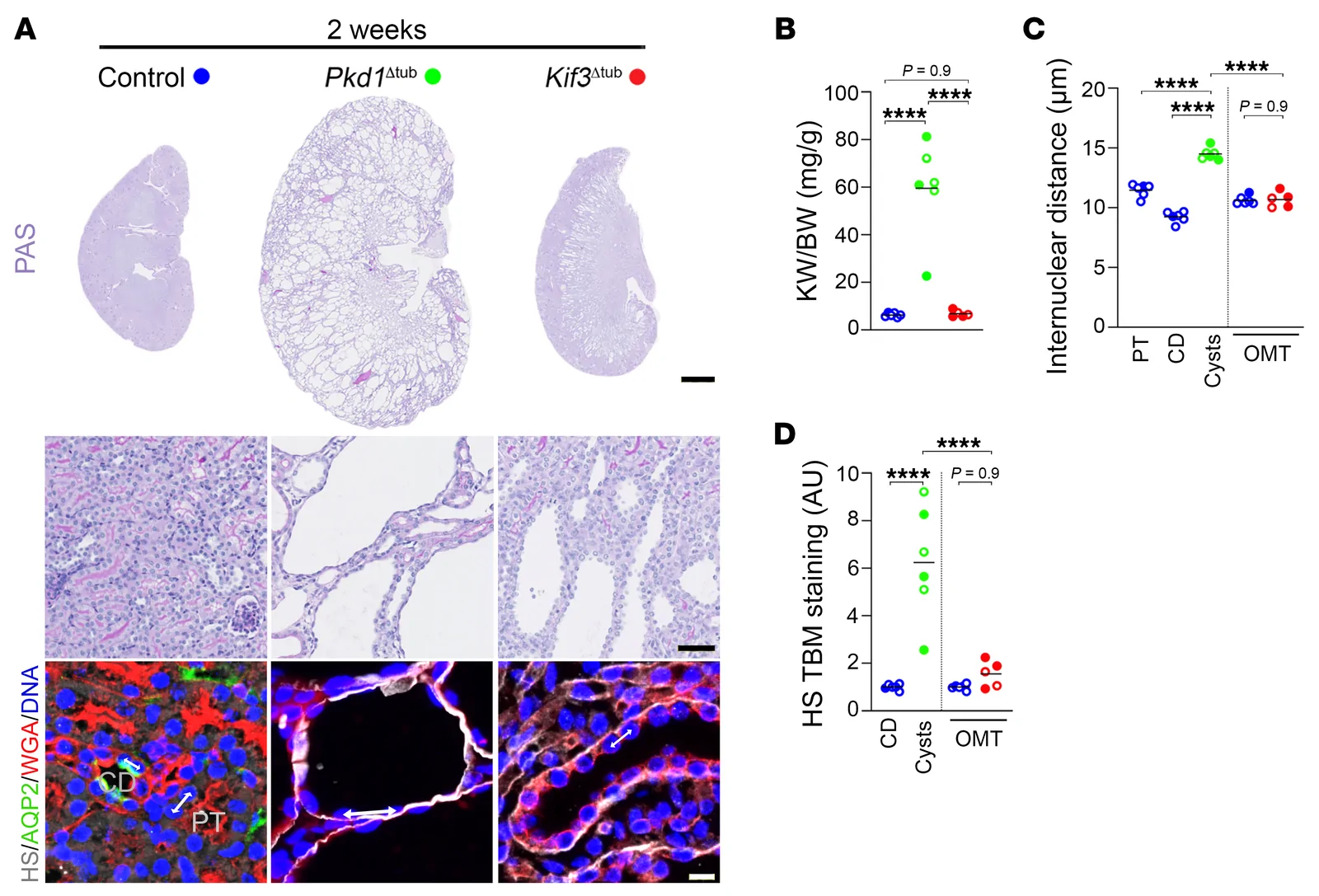
Comparison between neonatal cilia and *Pkd1* loss reveals distinct modes of tubule dilation. (**A**) PAS staining and labeling of HS, DNA, WGA, and AQP2 of kidneys from 2-week-old control, *Pkd1*^Δtub^, and *Kif3a*^Δtub^ mice. White arrows: examples of internuclear distance measurements. Scale bars: 1 mm (top), 50 μm (middle), 10 μm (bottom). (**B**–**D**) Quantification of KW/BW (**B**), internuclear distance (**C**), and HS staining (**D**) in the TBM of PTs, CDs, *Pkd1*^Δtub^ cysts, and outer medullary tubules (OMTs) in kidneys from the same groups of mice, as indicated. Each dot represents 1 female (closed circle) or male (open circle) mouse. One-way ANOVA followed by Tukey-Kramer test: ****P < 0.0001, or the indicated *P* value. *Pkd1^fl/fl^* or *Kif3a^fl/fl^* littermates lacking cre or rtTA transgene were used as control.

Single cilia-deficient mouse models typically develop cystic kidney disease resembling the ADPKD phenotype (24, 25). Cyst formation occurs rapidly when cilia are ablated during kidney development (24), but the cystic phenotype is drastically delayed when cilia are ablated at later time points (25). With the strategy that we used for cilia ablation, cysts have only been detected in 9-month-old male mice (26). Indeed, contrary to single *Pkd1* mutant mice and similarly to double *Pkd1*/cilia mutant mice, single *Kif3a* mutant mice did not show any spontaneous tubular distension or TBM thinning at 8 weeks. Proliferative index, internuclear distance, and TBM thickness remained unchanged after *Kif3a* inactivation in both proximal and distal tubules (Figure 3, A–F). We next conducted the same analyses in the context of neonatal tubular inactivation of *Pkd1* or *Kif3a*, as cilia ablation in the developing kidney leads to rapid cyst formation (Figure 4, A–D). Lactating mothers at birth were fed doxycycline, which induced explosive and diffuse cystogenesis in 2-week-old *Pkd1*^Δtub^ neonates, with increased tubule distension and marked HS accumulation in the TBM (Figure 4, A–D). In contrast, in single cilia-deleted neonates (*Kif3a*^Δtub^), the same treatment led to only a modest dilation of outer medullary tubules, with unchanged internuclear distance (Figure 4, A–C). *Kif3a*-deficient dilated tubules showed a mild, yet nonsignificant, increase in HS labeling compared with the outer medullary tubules of control littermates (Figure 4, A and D). Collectively, these findings indicate that cyst formation after cilia ablation is not initially associated with tubule distension or marked HS enrichment of the TBM, suggesting that the mechanisms of cyst formation after PC1 or cilia ablation do not fully overlap.

### TBM remodeling and CD distension coincide with a specific transcriptional program.

**Figure 5:**
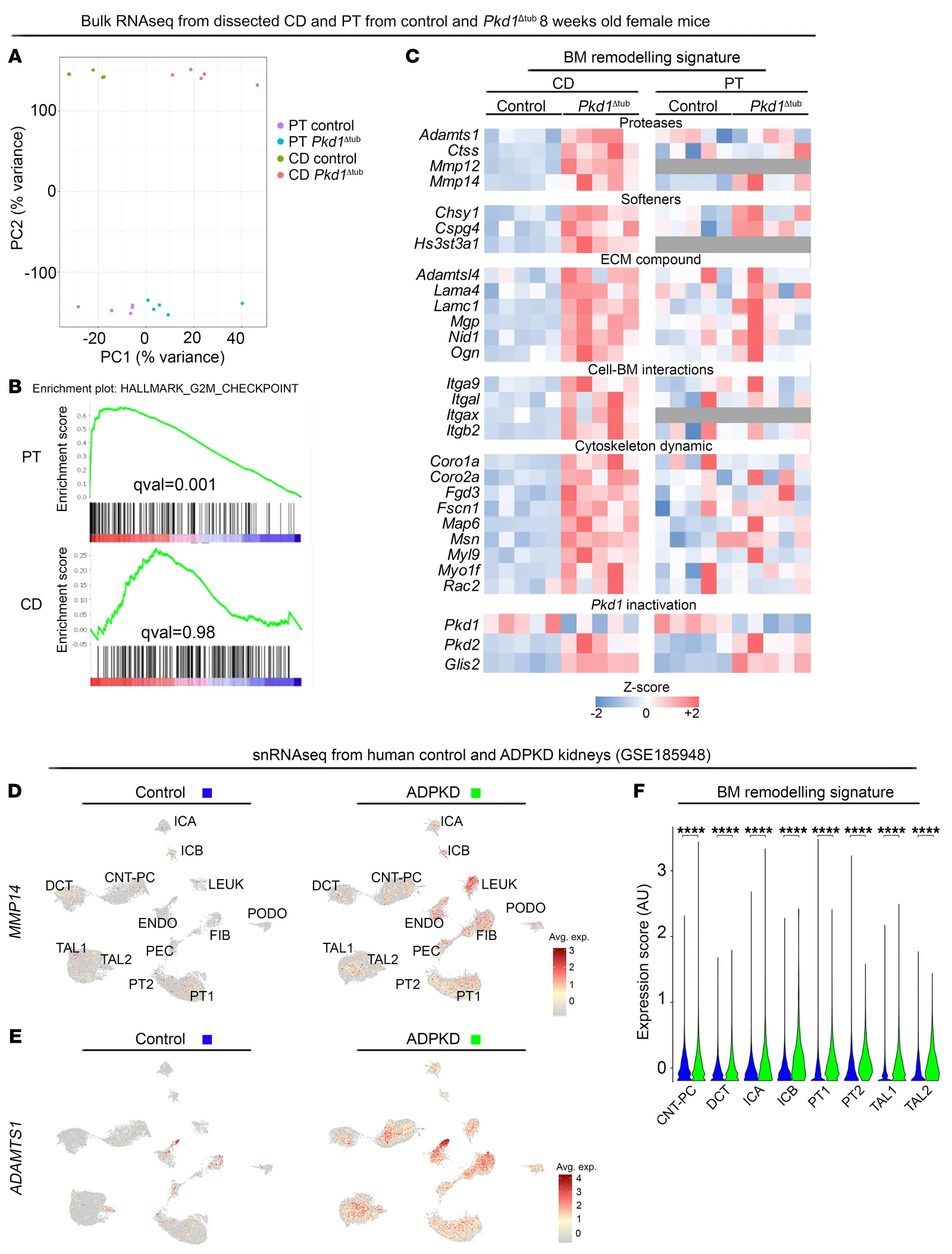
Transcriptomic profiling of microdissected PTs and CDs identifies a BM remodeling signature associated with tubule distension. (**A**) Principal component analysis of the transcriptome of PTs and CDs microdissected from control and *Pkd1*^Δtub^ 8-week-old female mice (i.e., 2 weeks after the end of doxycycline treatment; *n* = 5 mice per genotype; each dot represents a tubule segment from 1 mouse). (**B**) GSEA comparing proliferation-related gene sets between control and mutant mice in PTs or CDs. (**C**) Heatmaps showing the relative expression for TBM remodeling candidate genes in CDs and PTs microdissected from control and *Pkd1*^Δtub^ 8-week-old female mice (*n* = 5 mice per genotype). Gray in the heatmap of PT indicates undetectable expression. (**D** and **E**) Expression of *MMP14* (**D**) and *ADAMTS1* (**E**) in snRNA-seq data of control and ADPKD human kidneys. PEC, parietal epithelial cell; TAL, thick ascending limb of Henle’s loop; CNT-PC, connecting tubule and principal cell; ICA, type A intercalated cell; ICB, type B intercalated cell; PODO, podocyte; ENDO, endothelial cell; FIB, fibroblast; LEUK, leukocyte; URO, urothelium. (**F**) BM remodeling signature expression score in the indicated tubular cell population from control or ADPKD human kidneys. Bonferroni’s test: ****P < 0.0001. *Pkd1^fl/fl^* littermates lacking cre or rtTA transgene were used as control.

Our results suggest that cilia-dependent TBM remodeling promotes cyst formation in ADPKD and is decoupled from cell proliferation. Three nonmutually exclusive mechanisms can cause TBM distension without cell proliferation: (a) ECM digestion by proteases, which allows rapid BM remodeling during embryogenesis (27); (b) incorporation of BM softeners such as hydrating HS/CS, which allows rapid BM remodeling during *Caenorhabditis elegans* development (28); and (c) deformation of the BM by traction forces exerted by cells through cytoskeleton motor proteins, as documented during cancer invasion (29, 30). To gain insights into the effectors involved, we profiled the transcriptome of PTs and CDs microdissected from 8-week-old control and *Pkd1*^Δtub^ mice (Figure 5, A–C, and Supplemental Figure 5A). PT- and CD-specific gene expression profiles confirmed dissection quality (Supplemental Figure 5B). *Pdgfrb* and *Itgam* expressions were undetectable in CD, indicating the absence of contamination of the extracts by fibroblasts or macrophages, respectively. Overall, *Pkd1* inactivation induced a significant dysregulation in the expression of 2,151 genes in CDs but only 1,198 in PTs (Supplemental Figure 5C). Enrichment analyses showed an enrichment in gene sets related to cell proliferation in PC1-deficient PTs, but not in CDs (Figure 5B), a finding consistent with our immunolabeling experiments. More importantly, we identified a panel of 26 genes, which are upregulated in PC1-deficient CDs but not in PTs and are involved in the 3 aspects of BM remodeling that we prespecified, despite similar reduction in *Pkd1* mRNA with secondary increases in *Pkd2* and *Glis2* expression (Figure 5C) (14). The panel includes 4 genes encoding ECM proteases, 3 genes allowing the production of BM softeners (e.g., enzymes involved in HS/CS biogenesis; *Hs3st3a1*, *Chsy1*), 6 additional ECM components, and 13 genes involved in cell–BM interactions or cytoskeleton dynamics. Further reanalysis of single-nucleus RNA-seq (snRNA-seq) datasets comparing human ADPKD and healthy kidneys (31) showed that the BM remodeling signature that we identified in distending CDs from *Pkd1*^Δtub^ mice was significantly enriched in ADPKD human tubular cells (Figure 5, D–F). Of note, in these datasets of terminal human ADPKD kidneys, TBM remodeling gene induction was not restricted to distal segments, suggesting a generalization of the process at late stages.

### Primary cilia participate in the induction of BM remodeling genes.

To gain insight into the mechanisms by which cilia elicit tubule distension, we first took advantage of the translatome dataset derived from ciliated and deciliated *Pkd1*^–/–^ precystic tubules (14). Although BM remodeling genes were mostly induced in the distal nephron, which modestly contributes to the RNA pulled down in this dataset, we were able to replicate our findings for a set of genes, a fraction of which (e.g., *Adamts1*, *Adamtsl4*, *Chsy1*, *Map6, Fgd3*) showed cilia-dependent regulation (Supplemental Figure 6).

**Figure 6:**
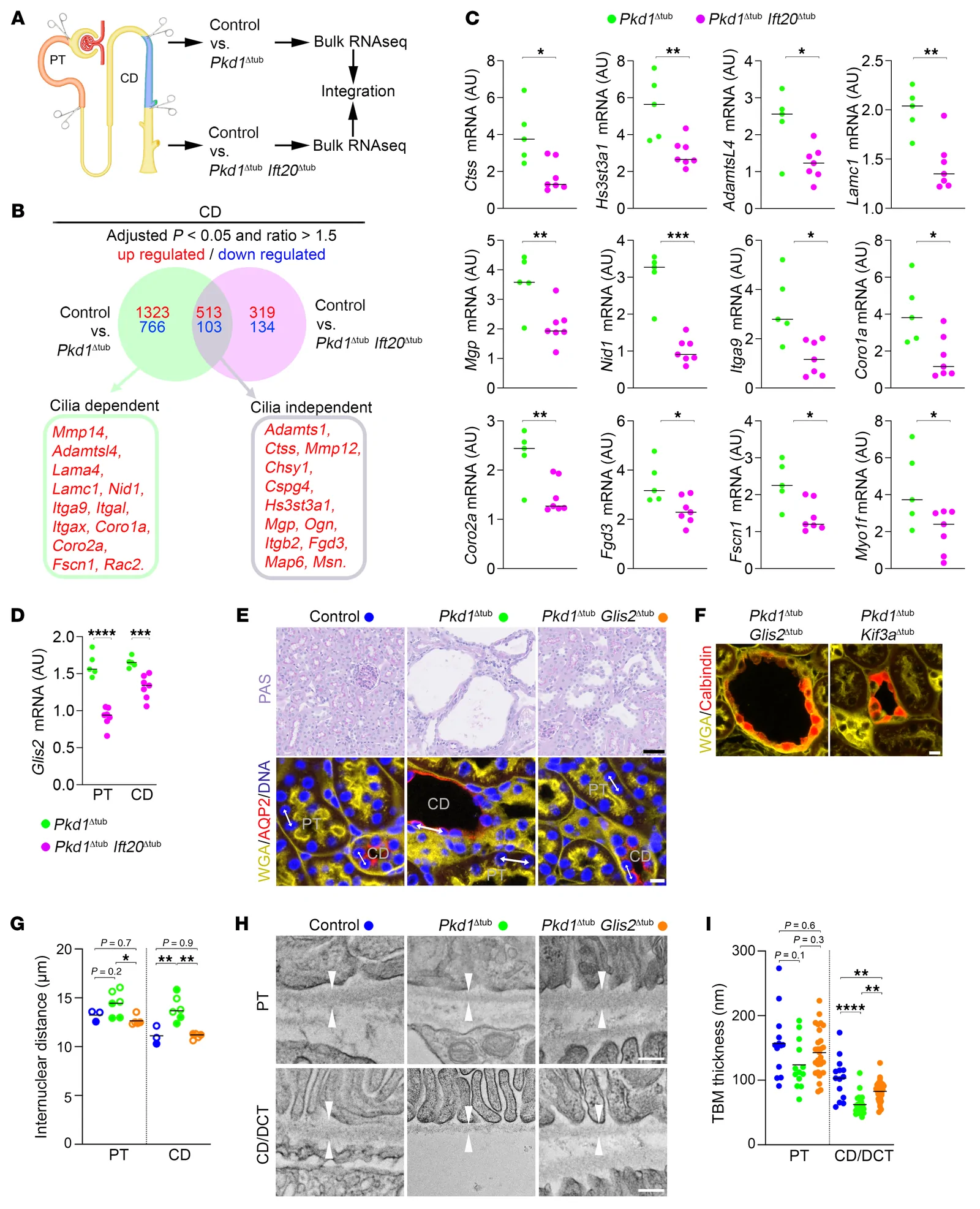
The primary cilium is instrumental in the induction of TBM remodeling genes downstream of PC1 loss and promotes tubule distension through GLIS2. (**A**) Schematic of the pipeline used to analyze cilia-dependent mediators of TBM remodeling: bulk RNA-seq was performed on CDs and PTs microdissected from control and *Pkd1*^Δtub^ *Ift20*^Δtub^ mice. Data were integrated with those obtained for *Pkd1*^Δtub^ (Figure 5C). (**B**) Venn diagram showing the number of genes with significant variation between *Pkd1*^Δtub^ and *Pkd1*^Δtub^ *Ift20*^Δtub^ mice and their corresponding littermate controls in CDs. The cilia-dependent and -independent genes of the BM remodeling signature are indicated below. (**C**) Dot plot showing the relative expression (fold increase compared with the relative controls) of the BM remodeling genes in ciliated and deciliated *Pkd1*-deficient CDs; only significant genes are shown. Each dot represents a tubule segment from 1 mouse. Student’s *t* test: *P < 0.05, **P < 0.01, ***P < 0.001. (**D**) Dot plot of *Glis2* mRNA expression in CDs or PTs from ciliated or deciliated *Pkd1*-deficient tubules. Each dot represents a tubule segment from 1 mouse. Two-way ANOVA followed by Šidák’s test: ***P < 0.001, ****P < 0.0001 (*P* value for interaction between genotype and segments = 0.0041). (**E**) PAS staining and labeling of PTs (WGA^+^) and CDs (AQP2^+^) of kidneys from 8-week-old control, *Pkd1*^Δtub^, and *Pkd1*^Δtub^ *Glis2*^Δtub^ mice. Arrows: examples of internuclear distance measurements. Scale bars: 50 μm (top), 10 μm (bottom). (**F**) Immunolabeling of calbindin (a marker of DCTs and early CDs) of kidneys from 8-week-old *Pkd1*^Δtub^ *Glis2*^Δtub^ and *Pkd1*^Δtub^ *Kif3a*^Δtub^ mice. Scale bar: 10 μm. (**G**) Quantification of mean internuclear distance in PTs and CDs of kidneys from 8-week-old control, *Pkd1*^Δtub^, and *Pkd1*^Δtub^ *Glis2*^Δtub^ mice. Each dot represents 1 male (open circle) or female (closed circle) mouse. (**H** and **I**) Transmission electron microscopy (**H**) and quantification of TBM thickness (**I**) in PTs, CDs, and DCTs in kidneys from the same groups of mice. Each dot represents 1 tubule (mean of 8–20 measurements per tubule; *n* = 3 mice per genotype). Scale bars: 200 nm. One-way ANOVA followed by Tukey-Kramer test: *P < 0.05, **P < 0.01, ****P < 0.0001, or the indicated *P* value. *Pkd1^fl/fl^* and *Pkd1^fl/fl^* *Glis2^fl/fl^* littermates lacking cre or rtTA transgene were used as control.

To complement this approach, we performed RNA-seq on PTs and CDs microdissected from *Pkd1*^Δtub^ *Ift20*^Δtub^ mice and control littermates (Figure 6A). Integration of these data with our previous RNA-seq results revealed that cilia ablation counteracted the majority of the transcriptional dysregulation triggered by *Pkd1* ablation in CDs and PTs (Figure 6B and Supplemental Figure 7). Specifically, approximately half of the BM remodeling genes upregulated upon *Pkd1* ablation were reduced in deciliated CD (Figure 6C), suggesting that cilia promote tubule distension through transcriptional regulation.

### The transcriptional regulator GLIS2 participates in TBM remodeling and Pkd1-deficient tubule distension, downstream of cilia.

The transcriptional regulator GLIS2 was recently identified as an important mediator of CDCA (14) and was correspondingly downregulated in deciliated *Pkd1*-deficient PTs and CDs (Figure 6D). To assess the role of GLIS2 in tubule distension, we studied *Pkd1*^Δtub^ and *Pkd1*^Δtub^ *Glis2*^Δtub^ mice. As previously described, *Glis2* inactivation suppressed the cystic enlargement of *Pkd1*-deficient kidneys (14). Although *Glis2* ablation drastically prevented tubule dilation, blinded examination of kidneys consistently revealed slight focal tubular dilation in the cortex of *Pkd1*^Δtub^ *Glis2*^Δtub^ kidneys that were not observed in cilia-deficient *Pkd1*^Δtub^ animals (Figure 6E). These tubules were generally heterogeneously labeled by a calbindin-specific antibody, suggesting a DCT or early CD origin (Figure 6F). Overall, *Glis2* ablation completely restored CD internuclear distance (Figure 6G) but only partially mitigated TBM thinning (Figure 6, H and I). Of note, in these mice housed in a different animal facility, there was only a trend toward proximal TBM thinning at this time point. Taken together, these results suggest that cilia regulate TBM remodeling through both GLIS2-dependent and -independent mechanisms.

### Loss of Pkd1 is associated with altered tubule mechanics.

**Figure 7:**
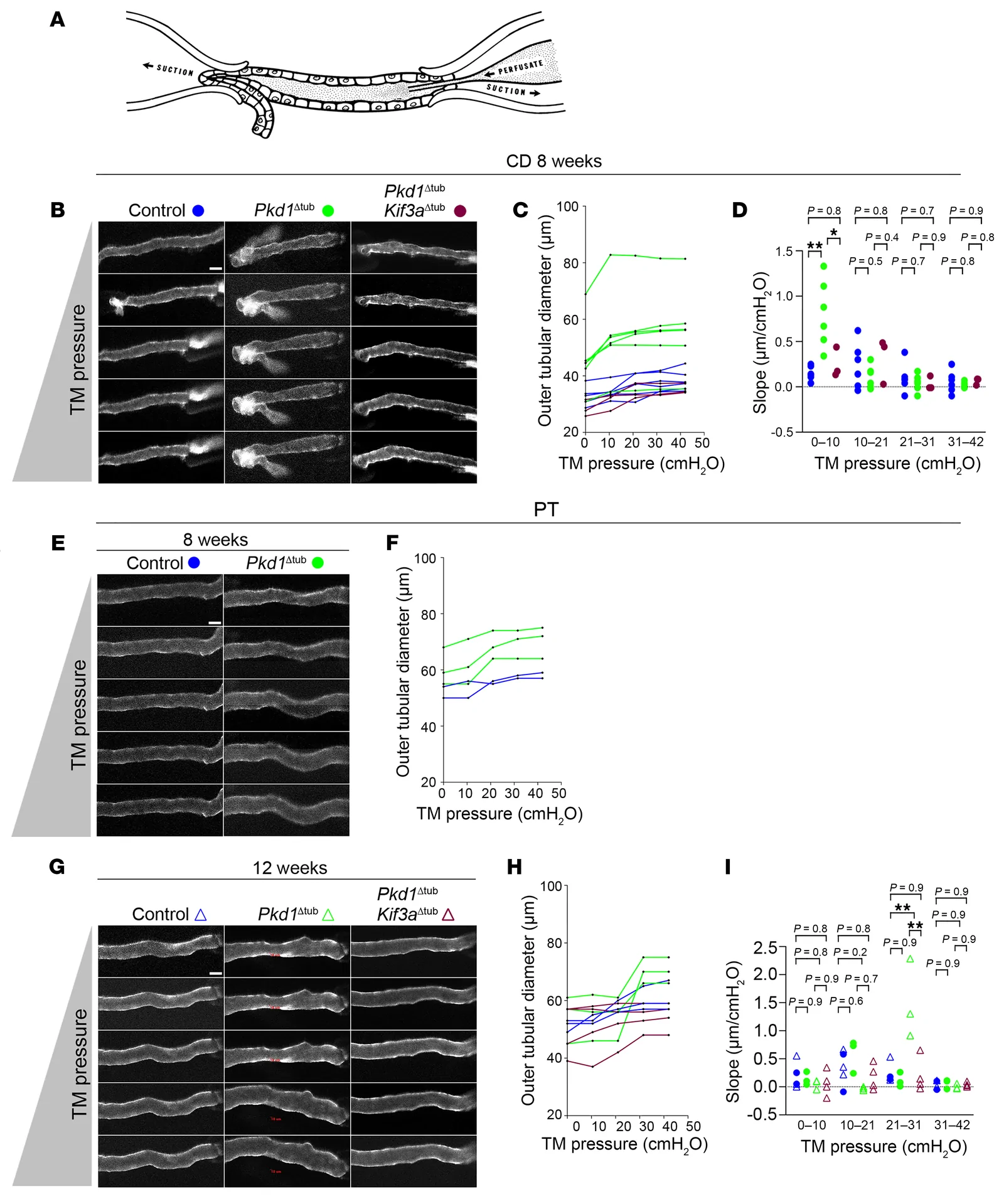
*Pkd1* deletion alters tubule biomechanics. (**A**) Scheme of isolated intact tubule perfusion setting. Adapted from *Journal of Clinical Investigation* (16). (**B** and **C**) Representative images (**B**) and quantification of the variation in outer tubule diameter induced by a progressive increase in transmural pressure (TM; **C**) in CDs isolated from 8-week-old control, *Pkd1*^Δtub^, and *Pkd1*^Δtub^ *Kif3a*^Δtub^ male mice. Each dot represents 1 tubule (mean of 10 measurements per tubule) for a pressure increment. (**D**) Quantification of the slope of the pressure-diameter curves for each pressure increment in CDs isolated from the same groups of mice. Each dot represents 1 tubule. Student’s *t* test: *P < 0.05, **P < 0.01. (**E** and **F**) Representative images (**E**) and quantification of the variation in outer tubule diameter induced by incremental increase in TM pressure (**F**) in PTs isolated from 8-week-old control and *Pkd1*^Δtub^ mice. Each dot represents 1 tubule (mean of 10 measurements per tubule) for a pressure increment. (**G** and **H**) Representative images (**G**) and quantification of the variation in outer tubule diameter induced by incremental increase in TM pressure (**H**) in PTs isolated from 12-week-old control, *Pkd1*^Δtub^, and *Pkd1*^Δtub^ *Kif3a*^Δtub^ mice. Each triangle represents 1 tubule (mean of 10 measurements per tubule) for a pressure increment. (**I**) Quantification of the slope of the pressure-diameter curves for each pressure increment in PTs isolated from 8- (circles) and 12-week-old (triangles) control, *Pkd1*^Δtub^, and *Pkd1*^Δtub^ *Kif3a*^Δtub^ mice. PTs from control animals at 8 (blue circle) and 12 weeks (blue triangle) were pooled in analyses. Each symbol represents 1 tubule. Scale bars: 50 μm. One-way ANOVA followed by Tukey-Kramer test: **P < 0.01. *Pkd1^fl/fl^* littermates lacking cre or rtTA transgene were used as control.

We asked whether remodeling of the TBM alters the physical properties of tubules. We isolated CDs and PTs from 8-week-old *Pkd1*^Δtub^ mice (with intact TBMs) and measured their diameter variations in response to increasing luminal pressure using a dedicated isolated tubule perfusion setup (Figure 7A) with physiologic pressure ranges (32). We observed that PC1-deficient CDs, which have undergone distension but not proliferation (i.e., dissected from 8-week-old mice; Figure 1, A–E), showed a steep increase in tubule diameter at low pressure (i.e., from 0 to 10 cmH_2_O; Figure 7, B–D). In contrast, PC1-deficient PTs, which have not yet undergone distension at this time point but are nonetheless larger than in control mice because of cell proliferation (Figure 1, A–E), showed similar pressure-diameter curves as control PTs (Figure 7, E and F). Repeating these experiments with PTs isolated from 12-week-old animals, a time point where distension has occurred in this segment (Figure 1, H and K), we found a similar steep increase in tubule diameter as in distended PC1-deficient CDs, although for a higher pressure threshold (i.e., from 21 to 31 cmH_2_O; Figure 7, G–I). Cilia ablation rescued the excessive dilation of PC1-deficient CDs (Figure 7, B–D) and PTs (Figure 7, G–I) by transmural pressure. Notably, decreasing pressure in distended PC1-deficient tubules resulted in a decrease of their diameter, indicating elastic deformation (Supplemental Figure 8). Together, these results demonstrate that tubule distension and TBM remodeling in ADPKD are associated with an alteration of tubule mechanics, characterized by a steep increase in tubule diameter with segment-specific pressure thresholds, which also appears as a cilia-dependent mechanism.

### Changes in TBM mechanical properties result in permissive conditions for cystogenesis.

**Figure 8:**
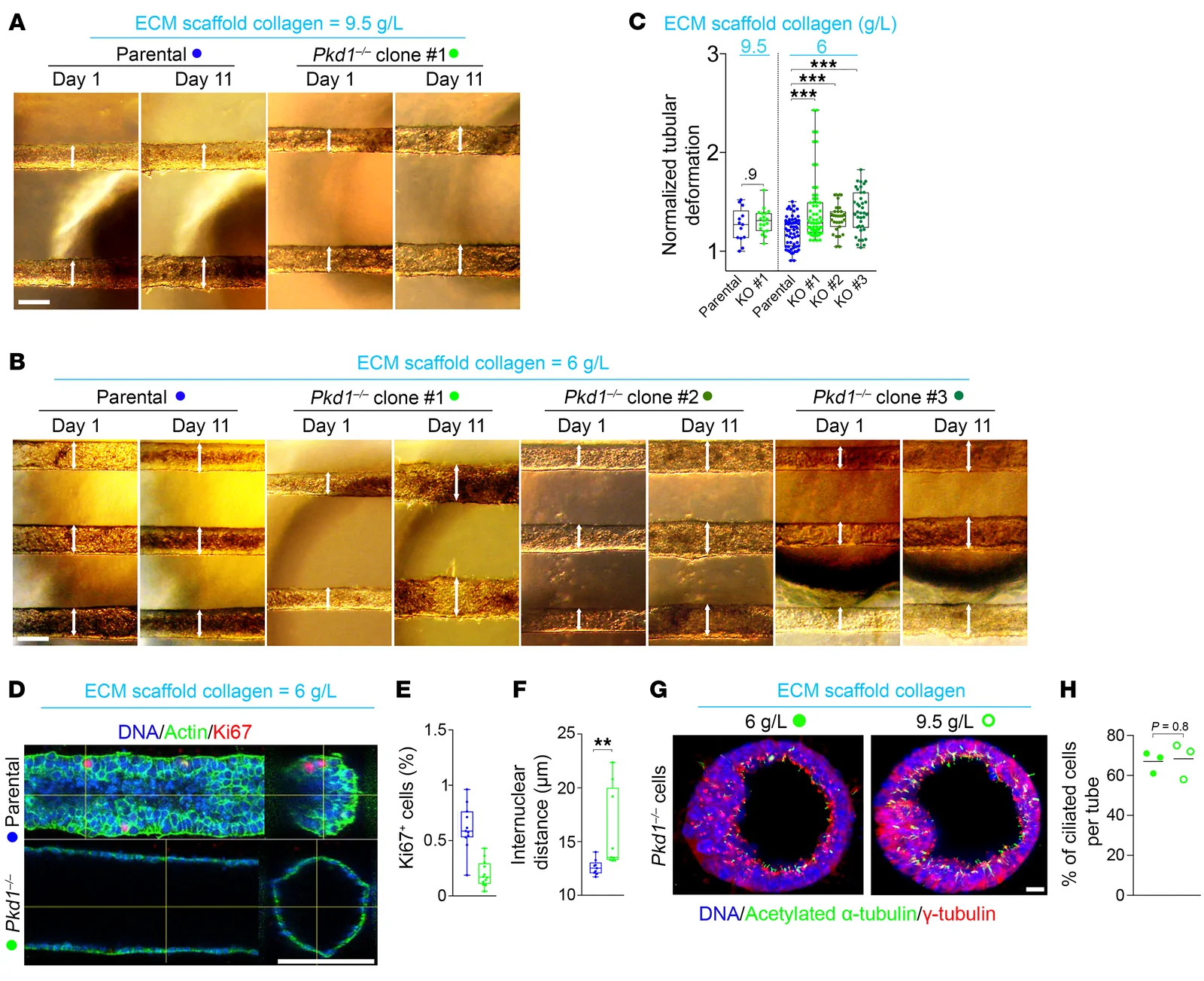
ECM mechanics modulate *Pkd1*^–/–^ tubule-on-chip dilation in vitro. (**A** and **B**) Representative images of tubules-on-chip formed by parental or *Pkd1*^–/–^ mIMCD3 clones in scaffolds containing 9.5 (**A**) or 6 (**B**) g/L collagen I, 1 and 11 days after confluency. Arrows indicate tubule diameter. Scale bars: 100 μm. (**C**) Quantification of the normalized tubule deformation in the corresponding tubules-on-chip at 11 days. Each dot represents 1 tubule from *n* = 8 for parental cells and *n* = 8, 5, and 4 chips for *Pkd1*^–/–^ clones 1, 2, and 3, respectively. (**D**–**F**) Labeling (**D**) and quantification of Ki67 (**E**) and internuclear distance (**F**) in parental and *Pkd1*^–/–^ tubules-on-chip. Each dot represents 1 tubule (parental cells: *N* = 3 chips/*n* = 9–11 tubules; *Pkd1*^–/–^ cells: *N* = 3 chips/*n* = 9–12 tubules). Mann-Whitney test: **P < 0.01. Scale bar: 100 μm. The box-and-whisker plots depict the minimum and maximum values (whiskers), the upper and lower quartiles, and the median. (**G**) Representative orthogonal sections of ciliated *Pkd1*^–/–^ tubules-on-chip embedded in 6 or 9.5 g/L collagen I and maintained under static conditions for 5 days. Cilia were identified by immunostaining of acetylated α-tubulin (green) and γ-tubulin (red). Scale bar: 100 μm. (**H**) Corresponding quantification of percentage of ciliated cells per tubule. Each dot represents 1 tubule (*n* = 3 tubules per condition). The reported *P* value was calculated using a Student’s *t* test. The data shown in this figure are derived from at least 2 different independent cell seeding experiments.

Our results regarding the early modifications of TBM in ADPKD suggest that increased ECM deformability favors cystogenesis. To investigate this question in vitro, we used an established tubule-on-chip model, whereby immortalized tubular cells form ciliated tubules of physiological dimensions in a deformable ECM scaffold (33). Using edited ciliated clonal lines derived from mouse collecting ducts (mIMCD3) (34), we observed only minor differences between control and *Pkd1*^–/–^ clones in the ability to dilate ECM scaffolds with a collagen content of 9.5 g/L (Figure 8A). However, by lowering collagen content, thereby reducing stiffness, we observed that *Pkd1*^–/–^ clones dilated the ECM scaffold to a larger extent than the parental line (Figure 8, B and C). This increased dilation was not caused by increased cell proliferation but by an increase in internuclear distance (Figure 8, D–F). These in vitro results demonstrate that ECM mechanics affect the ability of *Pkd1*^–/–^ cells to deform tubules, so that ECM deformability facilitates dilation. Ciliation of *Pkd1*-deficient cells was unaffected by collagen content (Figure 8, G and H), indicating that ECM mechanics do not regulate *Pkd1*^–/–^ tubule dilation by affecting ciliogenesis.

**Figure 9:**
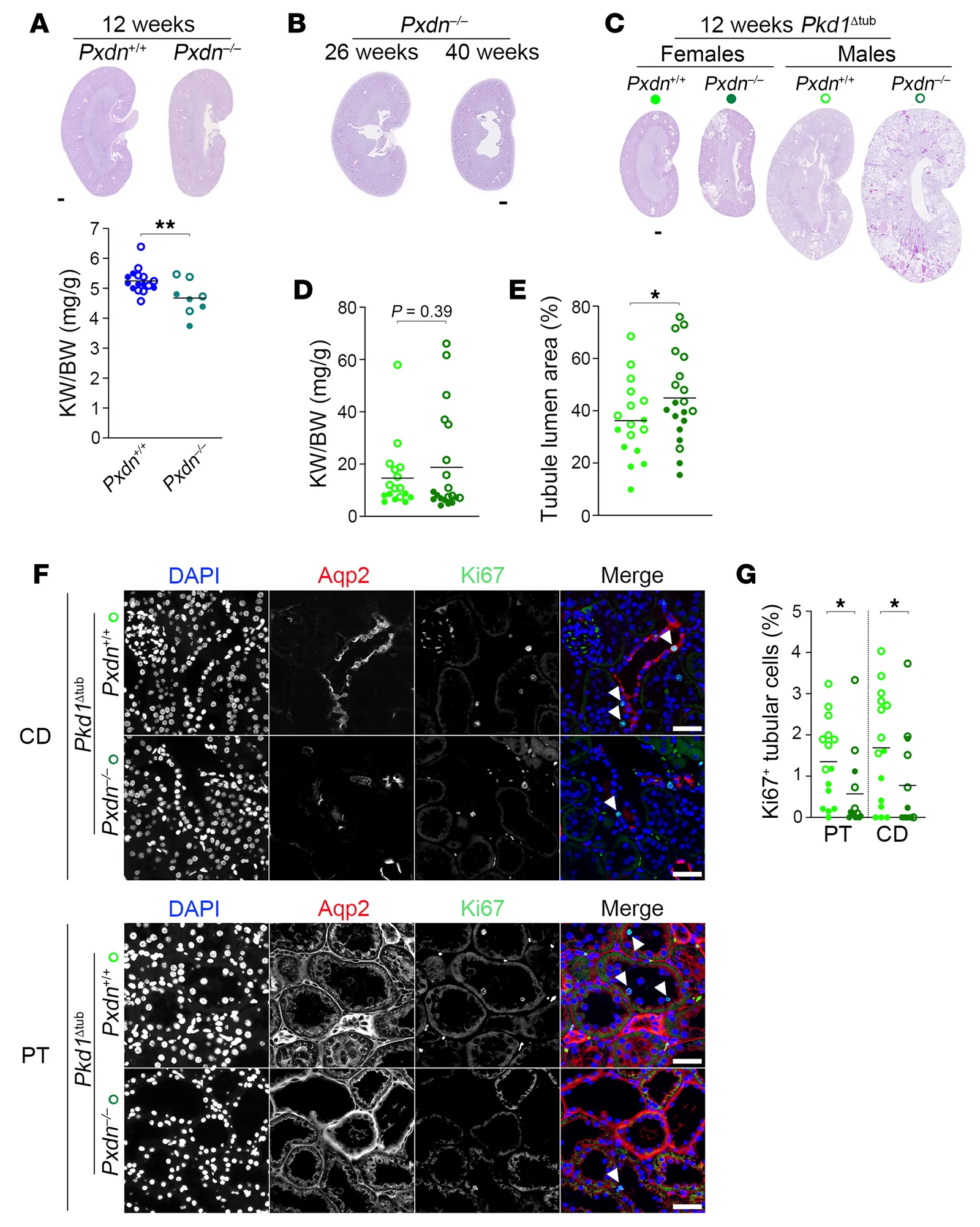
Decrease in TBM stiffness promotes cyst formation in vivo. (**A**) PAS staining and quantification of KW/BW of kidneys from 12-week-old control or *Pxdn^–/–^* female (closed circle) and male (open circle) mice. (**B**) PAS staining of kidney sections from aged *Pxdn^–/–^* mice showing no cyst development. (**C**–**E**) PAS staining (**C**), quantification of KW/BW (**D**), and tubular dilations (**E**) of kidneys from 12-week-old *Pkd1*^Δtub^ female (closed circle) and male (open circle) mice with or without concomitant *Pxdn* gene inactivation. Scale bars: 0.5 mm (**A**–**C**). For **A**, **D**, and **E**, each dot represents 1 mouse. *P* value indicates the *Pxdn* genotype effect in 2-way ANOVA followed by Tukey-Kramer test (sex and *Pxdn* genotype; *P* value for interaction between sex and genotype = 0.3 and 0.8 in **D** and **E**, respectively). *P < 0.05, **P < 0.01. (**F** and **G**) Labeling (**F**) and quantification of Ki67^+^ cells (**G**) in CDs and PTs in kidneys from the same groups of mice. Each dot represents 1 mouse. Scale bars: 40 μm. *P* values indicate the *Pxdn* genotype effect in 2-way ANOVA (sex and *Pxdn* genotype; *P* value for interaction between sex and genotype = 0.5 and 0.1 in PTs and CDs, respectively; *P < 0.05).

To test this concept in vivo, we inactivated *Pxdn*, which encodes peroxidasin, a collagen IV–specific cross-linking enzyme (in which germinal ablation induces a 20% decrease in TBM elastic modulus) (19), in *Pkd1*^Δtub^ mice. *Pxdn*^–/–^ single mutant mice have slightly smaller kidneys than control at baseline and do not spontaneously develop cysts, even in aging (Figure 9, A and B). The modest alteration of TBM mechanics caused by *Pxdn* inactivation translated into increased cyst burden in *Pkd1*^Δtub^ mice (Figure 9, C–E). At the studied time point, cystogenesis was more pronounced in males compared with females (*P* value for sex by 2-way ANOVA < 0.001). However, the impact of *Pxdn* inactivation on cyst area was not affected by sex (*P* value for interaction between sex and genotype by 2-way ANOVA = 0.8). These results are consistent with a model where reduced TBM stiffness facilitates tubule deformation and cystogenesis. While cyst formation was increased in *Pkd1*^Δtub^ *Pxdn*^–/–^ mice compared with *Pkd1*^Δtub^ mice, this did not translate into an increase in kidney weight/body weight ratio (KW/BW; Figure 9D). This discrepancy prompted us to assess tubular cell proliferation, which was overall reduced in the context of *Pxdn* inactivation (Figure 9, F and G), in line with the observation that stiffer substrates usually promote cell proliferation (35). The fact that reducing BM stiffness (via *Pxdn* inactivation) promoted tubule distension while reducing cell proliferation, further supports the view that, besides cell proliferation, BM mechanics and tubule distension are involved in cystogenesis.

### Tubule obstruction exacerbates PC1-deficient tubule distension and precipitates cystogenesis.

**Figure 10:**
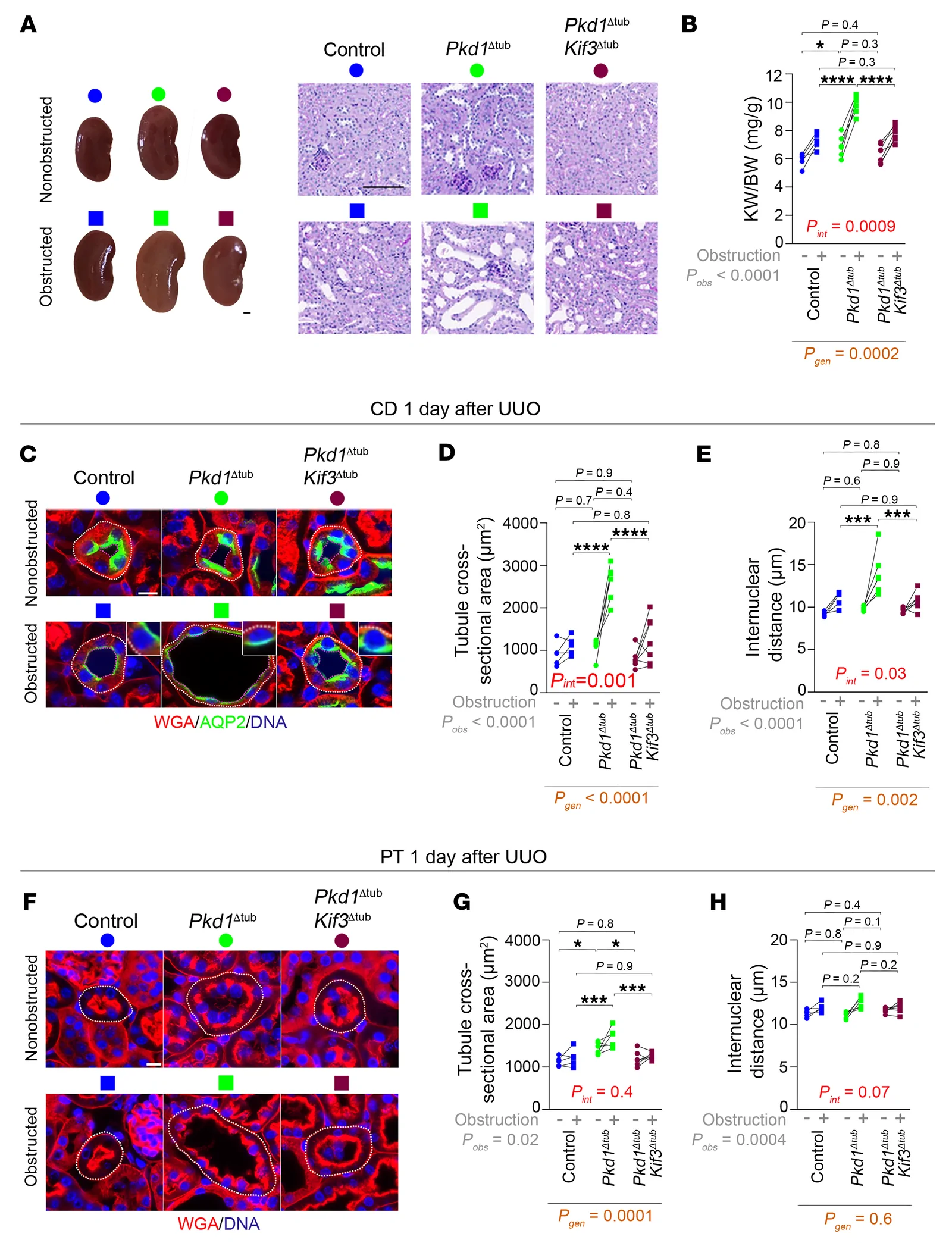
Acute ureteral obstruction accelerates *Pkd1*^Δtub^ distal tubule dilation in a cilia-dependent manner. (**A** and **B**) Kidney images and PAS staining (**A**) and quantification (**B**) of KW/BW of obstructed and nonobstructed kidneys from 8-week-old control, *Pkd1*^Δtub^, and *Pkd1*^Δtub^ *Kif3a*^Δtub^ 1 day after UUO. Scale bars: 1 mm (left), 0.1 mm (right). (**C**–**H**) Labeling of DNA, WGA (which stains the brush border of PTs and all BMs), and AQP2 (a CD marker; **C** and **F**) and quantification of mean tubule cross-sectional area (**D** and **G**) and internuclear distance (**E** and **H**) of CDs (**C**–**E**) and PTs (**F**–**H**) of kidneys from the same animals. Each pair of linked symbols (circle and square) represents the obstructed (square) and nonobstructed (circle) kidneys of an individual female mouse. Scale bars: 10 μm. Two-way ANOVA, *P* value for obstruction (gray; *P_obs_*), genotype (brown; *P_gen_*), and their interaction (red; *P_int_*), followed by Tukey-Kramer test: *P < 0.05, ***P < 0.001, ****P < 0.0001, or the indicated *P* value. *Pkd1^fl/fl^* or *Pkd1^fl/fl^* *Kif3a^fl/fl^* littermates lacking cre or rtTA transgene were used as control.

Tubular obstruction from enlarging cysts has been proposed to contribute to further cyst formation, and micropuncture and microdissection studies have shown that cysts frequently arise from obstructed tubules (36). We tested in vivo how alterations in TBM biomechanics affect tubule response to obstruction with respect to PC1 and cilia. After a single day of unilateral ureteral obstruction (UUO), *Pkd1*^Δtub^ mice showed a disproportional increase in kidney weight compared with controls. This was not the case in kidneys with concomitant cilia ablation (Figure 10, A and B, and Supplemental Figure 9A). The kidney weight increase correlated with enlargement of the CDs and DCTs, but not PTs, and was associated with a marked increase in internuclear distance, indicating tubule distension (Figure 10, C–H, and Supplemental Figure 9, B–D). BrdU labeling in *Pkd1*-deficient CDs did not significantly change following obstruction (Supplemental Figure 9, E and F), suggesting that early cyst expansion after UUO occurs largely independently of cell proliferation, though the limited sample size of this analysis warrants caution in interpretation.

Two-way ANOVA confirmed that a significant interaction between genotype and obstruction governs distal nephron distension (Figure 10, D and E, and Supplemental Figure 9, B–D). Of note, this first set of experiments was conducted on 8-week-old females, which at this age already display a thinner TBM in CDs but, contrary to males, have negligible tubule dilation at baseline. Similar results were obtained with male mice (Supplemental Figure 10).

**Figure 11:**
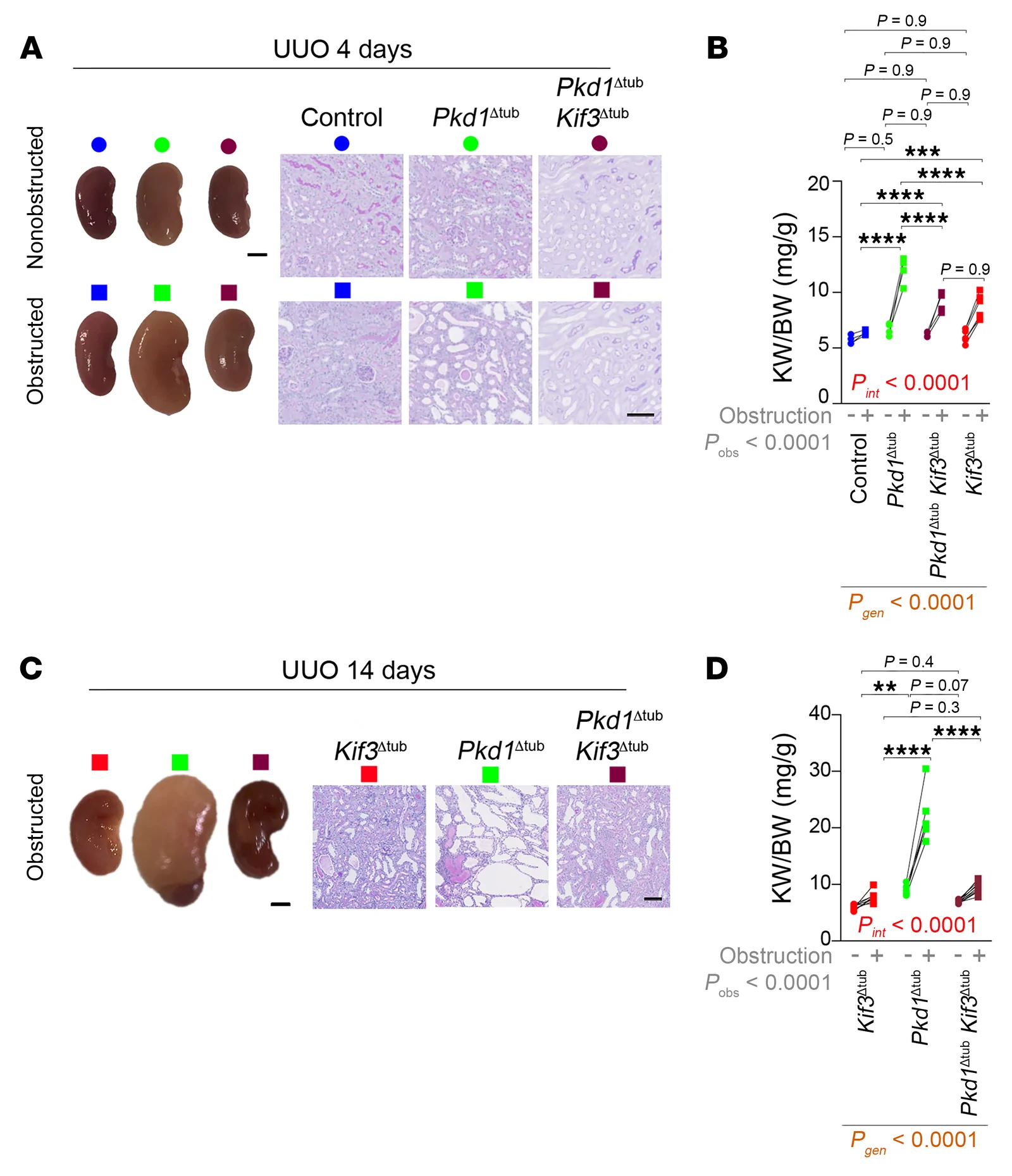
Ureteral obstruction triggers explosive cystogenesis in *Pkd1*^Δtub^ distal tubules in a cilia-dependent manner. (**A** and **B**) Kidney images and PAS staining (**A**) and quantification (**B**) of KW/BW of obstructed and nonobstructed kidneys from control, *Pkd1*^Δtub^, *Pkd1*^Δtub^ *Kif3a*^Δtub^, and *Kif3a*^Δtub^ mice (in red) 4 days after UUO performed at 8 weeks of age. (**C** and **D**) Kidney images and PAS staining (**C**) and quantification (**D**) of KW/BW of kidneys from *Kif3a*^Δtub^ and *Pkd1*^Δtub^ *Kif3a*^Δtub^ 14 days after UUO performed at 8 weeks of age. Single *Kif3a* mutant mice (*Kif3a*^Δtub^ mice) are presented to show apparent epistasis of *Kif3a* over *Pkd1* inactivation. Scale bars: 1 mm (kidney images), 0.1 mm (PAS). Each pair of linked symbols (circle and square) represents the obstructed (square) and nonobstructed (circle) kidneys of an individual female mouse. Two-way ANOVA, *P* value for obstruction (gray; *P_obs_*), genotype (brown; *P_gen_*), and their interaction (red; *P_int_*), followed by Tukey-Kramer test: **P < 0.01, ***P < 0.001, ****P < 0.0001, or the indicated *P* value. *Pkd1^fl/fl^* or *Pkd1^fl/fl^* *Kif3a^fl/fl^* littermates lacking cre or rtTA transgene were used as control.

**Figure 12:**
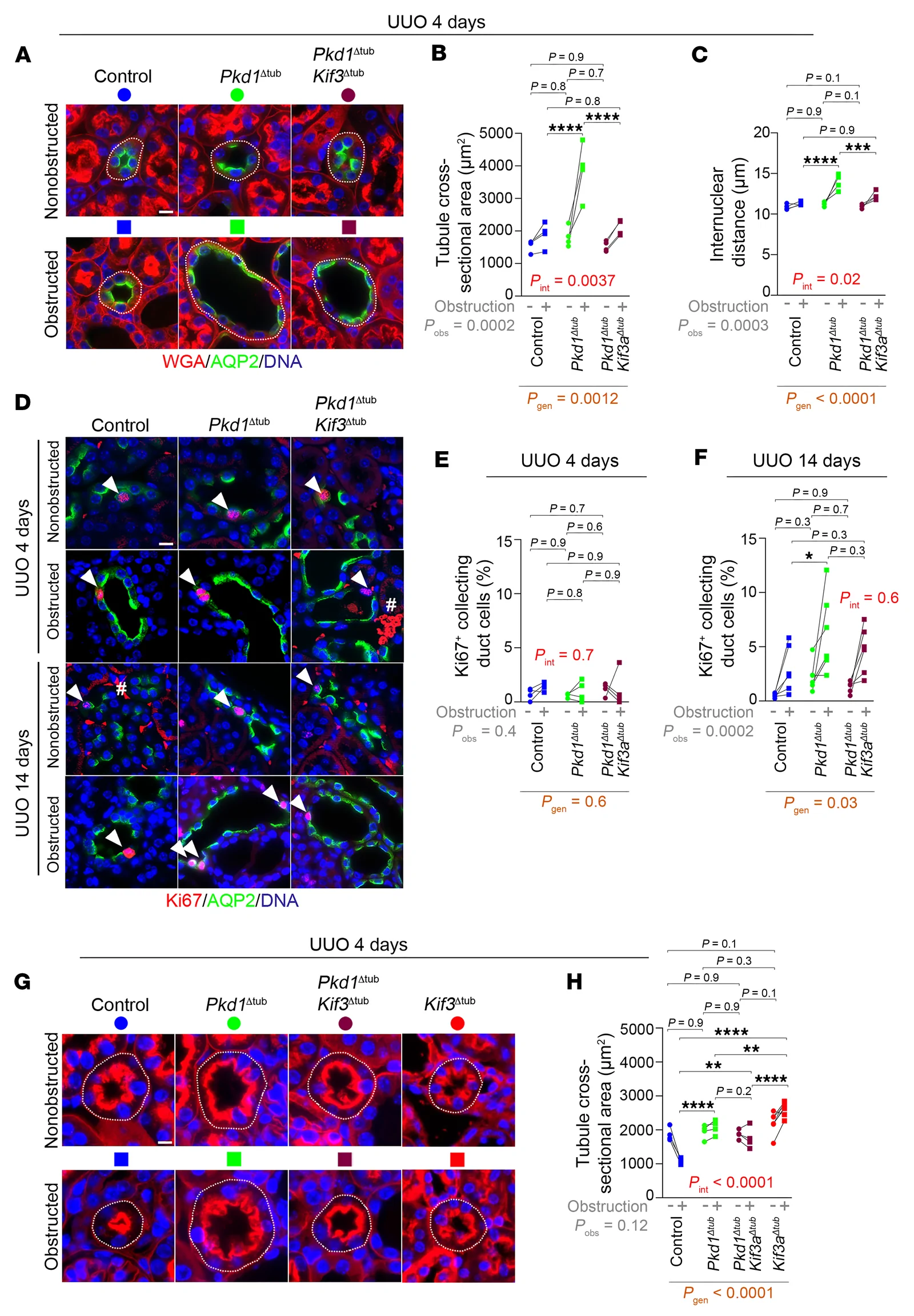
Accelerated cystogenesis after obstruction correlates with tubule distension and not proliferation. (**A**–**C**) Fluorescent labeling of DNA, WGA (which stains the brush border of PTs and all BMs), and AQP2 (a CD marker) (**A**) and quantification of CD mean tubule cross-sectional area (**B**) and mean internuclear distance (**C**) in kidneys from control, *Pkd1*^Δtub^, and *Pkd1*^Δtub^ *Kif3a*^Δtub^ 4 days after UUO performed at 8 weeks of age. (**D**–**F**) Fluorescent labeling of DNA, Ki67, and AQP2 (**D**) and quantification (**E** and **F**) of Ki67 staining (arrowheads; # indicates nonspecific red blood cell autofluorescence) in CDs of kidneys from control, *Pkd1*^Δtub^, and *Pkd1*^Δtub^ *Kif3a*^Δtub^ 4 (**E**) and 14 (**F**) days after UUO. (**G** and **H**) Representative images (**G**) and quantification of the PT cross-sectional area (**H**) in kidneys from control, *Pkd1*^Δtub^, *Pkd1*^Δtub^ *Kif3a*^Δtub^, and *Kif3a*^Δtub^ (in red) 4 days after UUO. Dashed lines in images indicate tubule perimeters. Scale bars: 10 μm. Each pair of linked symbols (circle and square) represents the obstructed (square) and nonobstructed (circle) kidneys of an individual female mouse. Two-way ANOVA, *P* value for obstruction (gray; *P_obs_*), genotype (brown; *P_gen_*), and their interaction (red; *P_int_*), followed by Tukey-Kramer test: *P < 0.05, **P < 0.01, ***P < 0.001, ****P < 0.0001, or the indicated *P* value. *Pkd1^fl/fl^* or *Pkd1^fl/fl^* *Kif3a^fl/fl^* littermates lacking cre or rtTA transgene were used as control.

Prolonging obstruction resulted in cystic transformation of PC1-deficient kidneys, with a 2- and 3-fold increase in KW/BW 4 and 14 days after the surgery, respectively (Figure 11, A–D, and Supplemental Figure 11, A and B). Cilia ablation reduced obstructed PC1-deficient kidney enlargement to the mild level observed in cilia-deficient mice with intact *Pkd1* (i.e., *Kif3a*^Δtub^; Figure 11, B–D). Cystic transformation of *Pkd1*-deficient kidneys was mainly the consequence of an increase in DCT and CD cross-sectional area, while tubule internuclear distance remained higher than in control or PC1-deficient DCTs and CDs lacking cilia (Figure 12, A–C, and Supplemental Figure 11, C–H). We then asked if the cystic transformation of PC1-deficient tubules could be caused by an excessive proliferative response to obstruction. As expected, labeling cycling cells with PCNA or Ki67 revealed that, with time, obstruction increased the fraction of cycling cells in CDs and DCTs. However, the magnitude of this increase was similar across genotypes (Figure 12, D–F, and Supplemental Figure 12, A–D). Consistent with our baseline findings, nonobstructed PTs lacking PC1 displayed a higher fraction of cycling cells than control or deciliated PTs. Obstruction further increased the cycling cell fraction in all genotypes (Supplemental Figure 12, C–E). The proliferation rate did not correlate with PT deformation: obstruction reduced PT cross-sectional area in control animals, indicating atrophy. This compaction of PTs was similarly prevented by *Pkd1* or cilia ablation (Figure 12, G and H).

In summary, the cystogenic response to obstruction corresponded with the observed effect of PC1 and cilia on TBM structure and mechanics: PC1-deficient CDs and DCTs have a thinner TBM and higher distensibility than PTs and correspondingly dilate more strongly, precipitating cystogenesis.

### Transient obstruction is sufficient to precipitate irreversible cystogenesis.

**Figure 13:**
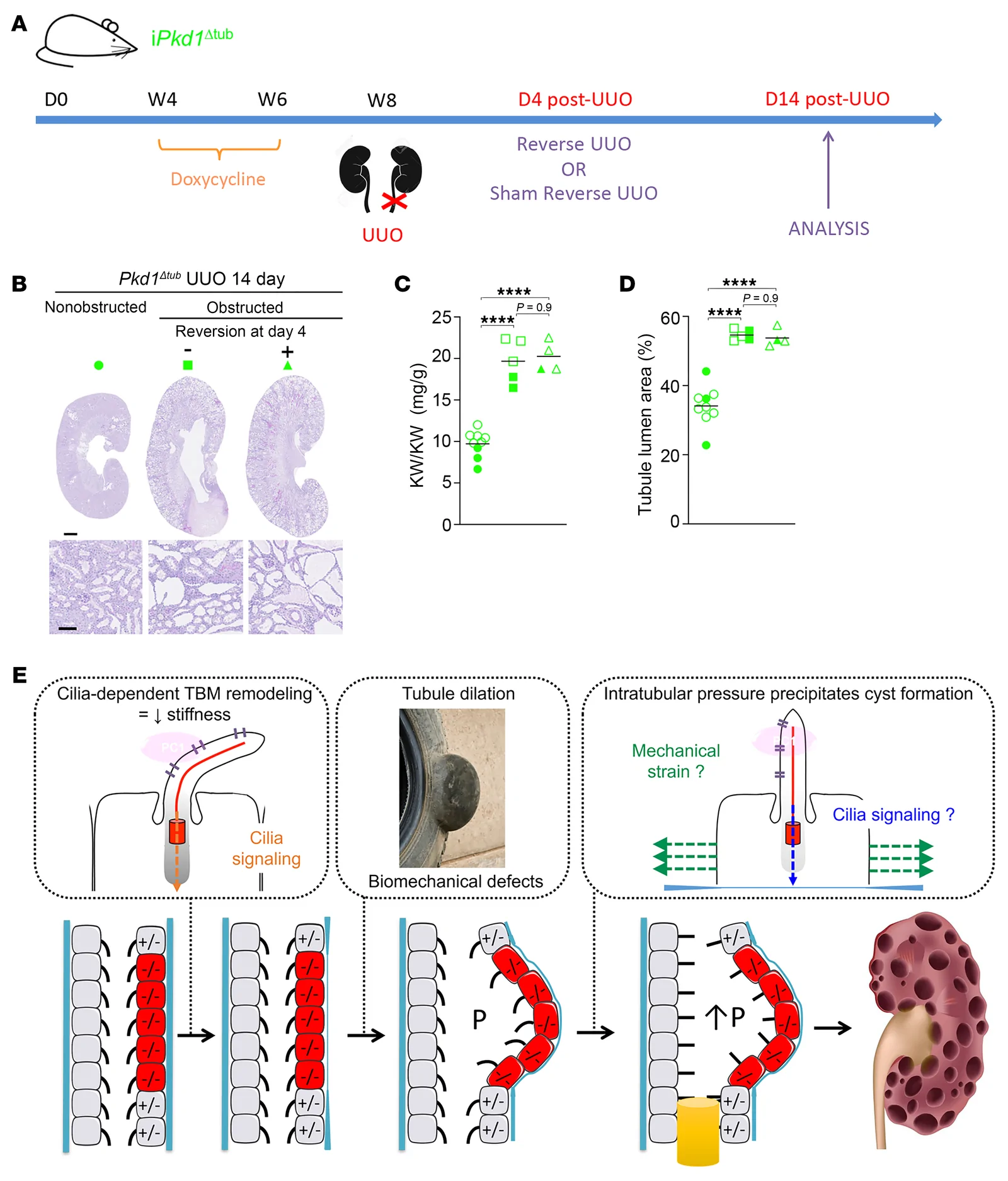
Transient obstruction triggers irreversible cystogenesis in *Pkd1*^Δtub^ mice. (**A**) Scheme of the protocol of R-UUO. Left kidney of 8-week-old *Pkd1*^Δtub^ mice was submitted to UUO, followed by a release of the obstruction (Reverse UUO) or a Sham operation (Sham Reverse UUO) 4 days after UUO, and mice were euthanized 10 days after the second surgery. (**B**–**D**) PAS staining (**B**) and quantification of KW/BW (**C**) and tubule dilations (**D**) of the kidneys from control or *Pkd1*^Δtub^ mice submitted to R-UUO or sham R-UUO. Scale bars: 2 mm (top), 0.1 mm (bottom). Each symbol represents an individual female (closed symbol) or male (open symbol) mouse kidney. One-way ANOVA followed by Tukey-Kramer test: ****P < 0.0001 or the indicated *P* value. (**E**) Proposed “tire bulge” model for cyst formation in ADPKD: PC1 loss triggers cilia-dependent remodeling of BM. BM remodeling in turn promotes tubule distension, which is exacerbated by transmural hydrostatic pressure (P). Transient tubule obstruction increases luminal pressure, tubule dilation, and tubular cell stretch, precipitating cystogenesis.

Previous reports showed that only a minority of PKD cysts are obstructed at any given time (22, 36), suggesting that permanent tubule obstruction is not a frequent driver of cystogenesis. To study the effect of a transient obstruction on cyst formation, we used a reversible UUO (R-UUO) model: *Pkd1*^Δtub^ mice underwent ligation of the distal ureter, followed 4 days later by either surgical release of the obstruction or a sham operation, and the kidneys were analyzed at 14 days (i.e., 10 days after UUO reversion; Figure 13A). Notably, release of the obstruction after 4 days failed to reduce cyst growth at 14 days (Figure 13, B–D), indicating that transient obstruction is sufficient to commit tubules to an irreversible cystogenic program.

Altogether, these results establish a TBM-centered model of cystogenesis in which *Pkd1* loss drives cilia-dependent remodeling of TBM shape and mechanics, which, in conjunction with increased luminal pressure, produces tubule dilation in a manner resembling the formation of a tire bulge (Figure 13E). In this condition of increased tension, obstruction precipitates cystogenesis independent of cell proliferation.

## Discussion

ADPKD is the most common ciliopathy. Although its genetics are well understood, the mechanism of its defining feature — cyst formation — has remained unclear. We show that cilia and PC1 regulate TBM composition, and thereby its biomechanical properties, and that disturbance of TBM shape and biomechanics drives tubular dilation and cyst formation, defining a cystogenic mechanism that we believe to be previously unrecognized in ADPKD.

TBM remodeling in cystic epithelia has traditionally been viewed as a secondary, passive event. Earlier reports of TBM thickening in PKD came from chemically induced rat models or terminal human ADPKD kidneys with marked fibrosis (37–39), and ECM stiffening has been proposed to drive proliferation via mechanosensitive YAP/TAZ activation (40). In contrast, our data show that TBM alteration is a very early morphogenic consequence of *Pkd1* inactivation, present at the onset of distal tubule dilation: TBM thinning precedes the increase in internuclear distance that marks the reversible columnar-to-flattened epithelial transition caused by *Pkd1*/*Pkd2* loss (41). Thinning is accompanied by progressive HS accumulation, and both changes are predicted to lower TBM stiffness. Accordingly, pressure-diameter curves of isolated tubules revealed a strong correlation between TBM thinning, tubule distension in vivo, and increased distensibility ex vivo, and increasing ECM deformability facilitated *Pkd1*^–/–^ tubule-on-chip dilation and cystogenesis in *Pkd1*^Δtub^ mice. These data indicate that TBM remodeling and the resulting change in tubule mechanics play an important role in cyst formation. Because our models used diffuse tubular *Pkd1* inactivation, the kinetics may differ from patients, in whom scattered *Pkd1*-deficient clones must expand before substantially deforming the TBM. Notably, our findings illuminate clinical observations: various collagen mutations have been linked to renal cysts, and combined *COL4A1* and *PKD2* mutations accelerate the ADPKD course (42–44).

TBM remodeling in PC1 deficiency requires cilia: 2 strategies preventing ciliogenesis — targeting the ciliary motor subunit *Kif3a* and the intraflagellar transport protein *Ift20* — consistently prevented TBM remodeling and tubule distension in PC1-deficient kidneys. Cilia are known to regulate ECM composition in mesenchymal cells such as chondrocytes, where they are embedded in and monitor the matrix. In the tubule, cilia instead protrude into the lumen and are thought to sense urine flow via PC1/PC2. Consistent with cilia matching TBM mechanics to hydrodynamic load, TBM thickness decreases along the tubule in parallel with hydrostatic pressure (45), whereas glomerular hyperfiltration is associated with TBM thickening (46). In some contexts, such as kidney development (24) or — in our study — prolonged obstruction, cilia ablation itself causes tubule dilation, suggesting cilia signaling helps maintain tubule diameter when luminal pressure rises. However, early distension with TBM remodeling and HS accumulation was specific to *Pkd1* deficiency, indicating at least partially distinct mechanisms. BM remodeling and primary cilia both contribute to development and cancer (47), so the cilia–BM connection may have implications beyond the kidney.

Our transcriptomic data point to 3 nonmutually exclusive mechanisms that may cooperate to drive TBM remodeling upon *Pkd1* loss: upregulation of matrix proteases, a shift toward a more compliant matrix composition, and altered epithelial traction forces. Converging evidence supports each arm: inactivating the TBM protease ADAMTS1 in a *Pkd1* model attenuates cyst growth (48), reducing epithelial traction through ROCK inhibition modestly lowers PKD burden (40, 49), and altering glycosaminoglycan composition by deleting xylosyltransferase 2 is sufficient to induce cystogenesis (50). Increasing collagen IV deformability alone (*Pxdn* inactivation) did not dilate WT tubules but amplified cyst formation under *Pkd1* deficiency, mirroring patients with collagen IV mutations that are permissive to, but not sufficient for, cysts (42–44). Together, these mechanisms likely act cooperatively to lower the mechanical barrier to cyst formation.

We further show that cilia ablation abrogates induction of the TBM transcriptional program and that GLIS2, a transcriptional effector of cystogenesis downstream of cilia, is partly responsible for TBM thinning. Other ciliary pathways appear unlikely or unresolved contributors: Hedgehog signaling has been experimentally excluded (51–53), WNT and PDGF-β promote fibrosis and TBM thickening rather than thinning (54, 55), and a role for ciliary calcium signaling remains contested (56–59). Thus, transcriptional regulation, partly via GLIS2, appears to be the main link between cilia and TBM remodeling, although the upstream ciliary regulators remain to be defined.

Our study also refines the role of cell proliferation in ADPKD. Although abnormal proliferation is integral to ADPKD and drives cyst growth (15, 60), we find it becomes a significant driver later than previously appreciated. Early on, TBM remodeling is the primary morphogenetic event, driving distal nephron distension independently of proliferation; only later do distension and proliferation occur concurrently. UUO experiments dissociated these processes: obstruction increased tubular proliferation across all genotypes, yet initial dilation progressed only in PC1-deficient DCTs and CDs, where it coincided with TBM remodeling and excessive cell stretching. By enlarging the area available for daughter cell intercalation, TBM remodeling may both reduce cell crowding — limiting extrusion and apoptosis — and increase stretching, which accelerates the G_2_/M transition (61).

Cysts frequently arise from obstructed tubules in ADPKD animal models, linking luminal obstruction to cystogenesis (36). Obstruction can stem from intraluminal crystals, particularly calcium oxalate, which accelerates cystogenesis in PKD models (62); granular casts of necrotic proximal tubule debris after ischemia-reperfusion injury (63); hyaline casts from dehydration; or compression of adjacent nephrons by expanding cysts, which is the proposed basis of the “snowball effect,” whereby cysts preferentially emerge near preexisting ones (64). These obstructive events are usually thought to accelerate cystogenesis by activating injury and inflammatory pathways (64). Our results suggest that the mechanical component may be at least equally important: although tubular injury occurred in all segments, cyst formation was restricted to segments undergoing TBM remodeling and excessive distension. Consistent with this, an extension of our tubule-on-chip shows that both loss of flow and increased intraluminal hydrostatic pressure independently promote *Pkd1*^–/–^ tubule dilation (65). Together, these findings provide a mechanistic framework for how obstruction drives cystogenesis in ADPKD.

Our animal models recapitulate a feature of human ADPKD — faster cyst growth in distal segments than in PTs — and provide a mechanical explanation: CDs and DCTs have thinner, HS-enriched TBMs, precociously activate TBM-remodeling genes upon PC1 loss, and undergo early distension with altered biomechanics, culminating in excessive cell stretching and accelerated cystogenesis after obstruction. That reducing matrix stiffness increases cyst formation partially contradicts the prevailing view that elevated ECM stiffness drives tubular proliferation and disease progression (40, 66); that view may still apply to late cyst growth, where pericystic fibrosis predominates.

This study has several limitations. Our principal model relies on global tubular *Pkd1* inactivation and does not reproduce the mosaic pattern of human ADPKD, in which biallelic *Pkd1* loss occurs in only a fraction of cells. Because we examined mainly *Pkd1* inactivation, our conclusions may not extend to other polycystic kidney diseases. We did not characterize the effect of obstruction on TBM structure and composition. While a recent study reporting that ADAMTS1 — one of the proteases identified in our screen — contributes to cyst growth (48) provides indirect support for the proteolytic arm, direct causal evidence for the compositional and traction force arms remains to be established. Finally, we did not analyze double cilia/*Pkd1* mutants beyond 18 weeks and therefore cannot fully distinguish delayed cyst onset from true suppression.

In summary, we show that PC1 and cilia regulate the composition and mechanical properties of the TBM. Our findings reveal what we believe to be a previously unappreciated role for primary cilia in TBM regulation and support a biomechanical model for cyst formation in ADPKD.

## Methods

### Sex as a biological variable.

Our study examined male and female animals, and sex dimorphic effects are reported.

### Mice.

Mice were housed at constant ambient temperature in a pathogen-free facility on a 12-hour day/night cycle and were fed ad libitum in an enriched environment. Breeding and genotyping were performed according to standard procedures. All mice were on a C57BL/6 (mixed J/N) background. *Pkd1*^fl/fl^ mice (B6.129S4-*Pkd1*^tm2Ggg/J^, The Jackson Laboratory), *Kif3a*^fl/fl^ mice (*Kif3a*^tm1Gsn^, C57BL/6 genetic background, provided by Peter Igarashi, Stony Brook University, Stony Brook, New York, USA), and *Ift20*^fl/fl^ mice (*Ift20*^tm1.1Gjp^, provided by Gregory Pazour, UMass Chan Medical School, Worcester, Massachusetts, USA) were crossed to Pax8rtTA (Tg[*Pax8*-rtTA2S*M2]1Koes, The Jackson Laboratory) and TetO-Cre (Tg[tetO-cre]1Jaw, The Jackson Laboratory) mice to create mice with inducible tubule-specific *Pkd1* knockout (named *Pkd1*^Δtub^), double *Pkd1* *Kif3a* knockout (named *Pkd1*^Δtub^ *Kif3a*^Δtub^), and double *Pkd1* *Ift20* knockout (named *Pkd1*^Δtub^ *Ift20*^Δtub^). *Kif3a*^Δtub^ mice were additionally analyzed and compared with *Pkd1*^Δtub^ mice for tubular morphometry and TBM remodeling. *Pxdn*-deficient mice (referred to as *Pxdn*^–/–^) were kindly provided by Miklos Geiszt (Semmelweis University, Budapest, Hungary) and were crossed with *Pkd1*^Δtub^ mice to generate *Pkd1*^Δtub^ mice with or without constitutive *Pxdn* inactivation (named *Pkd1*^Δtub^ *Pxdn*^–/–^). *Pkd1*^Δtub^ *Glis2*^Δtub^ mice were provided in-house. Most animals received doxycycline hyclate (Abcam, ab141091, 2 mg/mL) in drinking water supplemented with 5% sucrose and protected from light, from postnatal day 28 to 42 to induce the inactivation of floxed alleles. In some experiments, doxycycline was administered from postnatal day 0 to 14 to induce neonatal inactivation of *Pkd1* or *Kif3a*. Animals lacking either TetO-Cre or Pax8rtTA were used as controls. Animals of both sexes were used in experiments. All studied animals were included in the analyses. To assess Cre recombination efficiency, *Pkd1*^Δtub^ mice were crossed with mice carrying the *iSuRe-Cre* reporter allele, which induces the expression of a membrane-localized tandem tomato (MbTomato) fluorescent protein upon cre-mediated recombination (67). These mice received doxycycline from postnatal day 28 to 42, and tomato expression in tubules was assessed at postnatal week 8.

### Use of artificial intelligence.

During revision of this manuscript, the authors used Claude (Anthropic; Claude Opus 4, model claude-opus-4-8) to assist with condensing the text to meet the journal’s word limit, harmonizing gene symbol formatting, and reorganizing methodological content into the Supplemental Methods. The tool was used under direct author supervision; it did not generate, analyze, or interpret any experimental data, and all scientific content, conclusions, and final wording were reviewed and approved by the authors, who take full responsibility for the integrity of the work.

### Statistics.

Differences between the experimental groups were evaluated using 1- or 2-way ANOVA followed, when significant (*P* < 0.05), by the Tukey-Kramer test. When only 2 groups were compared, unpaired *t* test or Mann-Whitney test was used as appropriate. In some experiments (as indicated in the figure legends), *Pkd1*^Δtub^ *Kif3a*^Δtub^ and *Pkd1*^Δtub^ *Ift20*^Δtub^ mice were pooled for analysis, as both genotypes disrupt ciliogenesis and produced indistinguishable phenotypes in our hands. Control and tubule-specific mutants were housed in the same cages. No randomization was performed. Determination of sample size was based on laboratory experience. We did not perform a priori sample size calculation. Quantification of TBM thickness and morphometric analysis were performed in a blind fashion. Linear regression was also used to correlate tubule cross-sectional area and internuclear distance in the CDs and DCTs at 8 weeks. The statistical analysis was performed using GraphPad Prism V10 software.

### Study approval.

All mouse experiments were conducted in accordance with the guidelines of the French government animal welfare policy. Animal procedures were approved by the ethical committee of the Ministère de l’Enseignement Supérieur, de la Recherche et de l’Innovation (APAFIS agreements 201907041733347, 2020090715389782, and 2020111915598007).

### Data availability.

The transcriptomic data supporting the findings of this study will be openly available in the public domain through the BioStudies database (accession number S-BSST2959). Additional RNA-seq datasets were derived from the NCBI Gene Expression Omnibus (GSE185948 and GSE232556). All the mice models used in this study can be obtained upon request to the corresponding author, with the agreement of the scientist who generated the initial transgenic line. Values for all data points in graphs are reported in the Supporting Data Values file.

A full description of the experimental procedures is provided in the supplemental materials.

## Author contributions

MM, SC, FT, AV, and FB designed the study. MM, B Lapin, RA, JV, AV, MB, PH, LC, GC, VS, AA, MCV, B Lelongt, ET, KO, SC, CS, and FB performed experiments. TB and MK provided mIMCD3 cells. MR and SS provided material from *Glis2*-deficient mice and intellectual insights. RB provided the *iSuRe-Cre* mice and revised related work. MM, FB, AV, RA, CC, B Lapin, JV, SD, and SC analyzed data. NC performed bioinformatic analysis of RNA-seq data. SG performed U-Net segmentation analysis. MM and FB drafted and revised the manuscript with the help of AV, SC, EWK, MK, SS, and FT. All authors approved the final version of the manuscript.

## Conflict of interest

The authors have declared that no conflict of interest exists.

## Funding support

- Agence Nationale de la Recherche (ANR; ANR-19-CE14-0016, ANR-23-CE14-0025-03, and ANR-25-CE13-4676).
- EMBO and ERA-EDTA fellowship program (ALTF927-2013 to FB and ALTF84-2011 to AV).
- “Investissement d’Avenir” program implemented by ANR (ANR-18-IdEx-0001, program Emergence; Emergence RM2724IDX248_MechaBaCil; to FB).
- Société Française de Néphrologie, Dialyse et Transplantation (D24025KK).
- Association pour l’Information et la Recherche sur les Maladies Génétiques Rénales.
- AMX grant (“Ministère de la Recherche et de l’Enseignement Supérieur”; to B Lapin).
- “Fondation pour la Recherche Médicale” grant (to MM [FDM201906008513], provided by the Durlach foundation; B Lapin [program FDT], and AV [ARF20150934110]).
- King Abdulaziz University grant (to RA).
- INSERM interface contract (to FB).
- European Commission FET-Open program (FETOPEN-01-2016-2017).
- “Association Polykystose France,” provided by the SFNDT.
- “Institut Pierre-Gilles de Gennes Laboratoire d’Excellence, Investissements d’Avenir” program (ANR-10-IDEX-0001-02 PSL, ANR-10-LABX-31, and ANR-11-LABX-0038).
- CNRS.
- INSERM.
- Université Paris Cité
- Sorbonne Université.
- Institut Curie.
- DFG KU1504/8-1 and DFG KU1504/9-1 (to EWK).
- DFG SFB1453 (project 431984000; to CS).
- DFG SCHE 2092/6-1 (project 562400794) and DFG SCHE 2092/1-3 (project 241702976) (to CS).
- DFG Heisenberg program (project 501370692; to CS).

## Supplementary Material

Supplemental data

Supporting data values

## Version 1 06/30/2026

In-Press Preview

## Version 2 09/15/2026

Electronic publication
