# Supplementary material for: Cilia–to–basement membrane signaling is a biomechanical driver in models of autosomal dominant polycystic kidney disease
Source: J Clin Invest. 2026 Jun 30;136(18):e196814. doi: 10.1172/JCI196814 (PMC13574157; doi:10.1172/JCI196814)
Supplement: Supplementary file 1 [file jci-136-196814-s185.pdf]

Cilia to basement membrane signalling is a biomechanical driver in models of autosomal dominant polycystic kidney disease

SUPPLEMENTAL METHODS

Quantification of *Pkd1* allele deletion

The dissociation of mouse kidneys was performed as previously described (71), with minor modifications. Briefly, kidneys from 6-week-old control and *Pkd1*$^{\Delta tub}$ mice were perfused via the left ventricle with perfusion buffer prepared from Hanks' Balanced Salt Solution containing Ca^{2+}, Mg^{2+} and sodium bicarbonate (HBSS; Eurobio, Ref. L0612-500), supplemented with $MgCl_2$ (0.5 mM; Sigma, Ref. M1028), sodium acetate (1 mM, pH 7), and HEPES (20 mM), with the final pH adjusted to 7.4 using NaOH. This perfusion buffer was supplemented with collagenase B (2.5 mg/mL; Roche, Ref. 11088831001) and dispase II (1.2 mg/mL; Roche, Ref. 04942078001). Excised kidneys were minced into 1–2 mm fragments and transferred to 50 mL tubes containing digestion buffer prepared from the same base buffer supplemented with collagenase B (2 mg/mL) and dispase II (200 µg/mL). Samples were incubated at 37°C for 45 min, with gentle mechanical dissociation by pipetting up and down using a 1000 µL pipette every 15 min. Enzymatic digestion was quenched with 10 mL of 20% FBS. The cell suspension was centrifuged at 250 × g for 3 min at 4°C, washed once with PBS, and centrifuged again under the same conditions. The final pellet was resuspended in FACS buffer and sequentially filtered through 70 µm and 40 µm strainers (Falcon, Ref. 352350 and 352340, respectively) to obtain a single-cell suspension. The single-cell suspension was labeled with fluorescein-conjugated Lotus tetragonolobus lectin (LTL), a proximal tubule marker, and subjected to flow cytometry sorting after the addition of the viability dye Sytox Blue (S34857, Invitrogen). The efficiency of *Pkd1* allele recombination was then assessed in sorted proximal tubular cells by genomic DNA qPCR. Genomic DNA was extracted using the NucleoSpin Blood kit (Macherey-Nagel, Ref. 740951.50), according to the manufacturer's instructions. qPCR was

performed using iTaq Universal SYBR Green Supermix (Bio-Rad, Cat. No. 1725124). *Pkd1* primers were designed to amplify the region spanning exons 2 and 3, which are excised upon Cre-mediated recombination: forward 5′-ACCTGTCCCACAACCTACT-3′ and reverse 5′-GCAAACACGCCTTCTTCTAATG-3′. The relative abundance of the non-recombined *Pkd1* allele was normalized to the reference loci β-actin (Actb; forward 5′-GTGACGTTGACATCCGTAAAGA-3′, reverse 5′-CCTCACCAAGCTAAGGATGC-3′) and Hk2 (forward 5′-GCCAGCCTCTCCTGATTTTAGTGT-3′, reverse 5′-GGGAACACAAAAGACCTCTTCTGG-3′), and reported as fold change relative to control mice.

Animal procedures

Euthanasia of mice consisted of an intraperitoneal injection of anesthetizing solution (ketamin 160 mg kg-1 and xylazin 8 mg kg-1) followed with cervical dislocation once the depth of anaesthesia was confirmed.

Unilateral ureteral obstruction (UUO) was performed in 8-week-old animals as previously described(68). Briefly, mice received an intraperitoneal injection of anesthetizing solution (ketamin 160 mg.kg-1 and xylazin 8 mg.kg-1) associated with intraperitoneal injection of buprenorphine (0.1 mg.kg-1) to prevent postoperative pain. Under optical microscope, a left-side laparotomy was performed. The left ureter was exposed and ligated with a nonabsorbable 6/0 black braided silk suture. Reversion of ureteral obstruction was performed as previously described (69). A midline laparotomy was made to expose the left ureter. Firstly, the left ureter was ligated twice with a nonabsorbable 6/0 black braided silk suture. A longitudinally split sterile silicone tube (soft walled silicone plastic tubing) was then gently placed around the ureter. At last, a ligature around the tube was made twice to anchor and maintain the tube closed before closing the incision. Four days after the first surgery of UUO, the abdominal wall was re-opened. The silicon tube was removed and the left ureter was cut between the two sutures. The superior part of the ureter was then re-implanted

within the bladder. Ureter section and reimplantation steps were skipped for sham reimplantation surgery.

Standard morphological analysis

For histopathological analysis, mouse kidneys were fixed in 4% paraformaldehyde for 24 hours at 4°C and embedded in paraffin. Four micrometers thick sections were stained with Periodic acid-Schiff (PAS). PAS-stained, full-size kidney sections were imaged using a whole-slide scanner Nanozoomer 2.0 (Hamamatsu) equipped with a 20X /0.75 NA objective coupled to NDP.view software (Hamamatsu). Quantification of tubule dilation was performed on PAS-stained whole kidney sections with ImageJ2 software Version 2.9.0/1.53t, using NDPITools Plugin (version 1.8.3)(70). Briefly, this plugin automatically split the original PAS kidney sections into several images at magnification 20X. Cortical fields were then manually selected across the renal cortex on each image. The tubular lumen surface was automatically measured in each extracted cortical field, as previously described (71), and the results were finally expressed as percentages of total cortical tubular lumen area of the selected fields.

Fluorescent kidney staining

Four micrometers-thick sections of paraffin-embedded kidneys were submitted to deparaffinization and hydration. Kidney sections were stained with the molecules listed in Supplemental Table 1. We used distinct antigen retrieval: incubation in 10mM Tris Base, 1mM EDTA Solution, 0.05% Tween 20, pH 9.0 or citrate buffer pH6 (Zytomed, ZUC028) for 15 minutes in high pressure condition in a TintoRetriever® pressure cooker (BioSB) for Wheat Germ Agglutinin (WGA), Aquaporin 2 (AQP2), Calbindin (CALB), Ki67 and Proliferating Cell Nuclear Antigen (PCNA) labelling or in 50 mM Tris base, 1 mM EDTA solution, 0.01% proteinase K (Roche, 3115836001) for 2 minutes at room temperature, followed by avidin/biotin blocking (Vector Laboratories, SP-2001) for

collagen IV, laminin and HSPG labelling (72). Then, kidney sections were incubated with primary antibody overnight at 4°C, followed with the appropriate Alexa Fluor-conjugated secondary antibody. Images were acquired using the Zeiss Apotome 2 CO2 at the original magnification of 40X or Zeiss Spinning Disk L3 at the original magnification of 63X, and processed with ImageJ2 software.

BrdU incorporation assay

Six hours following UUO, control, *Pkd1*$^{\Delta tub}$ and *Pkd1*$^{\Delta tub}$; *Kif3a*$^{\Delta tub}$ mice received a single intraperitoneal injection of BrdU (100 mg/kg) and were sacrificed 18 hours after BrdU injection (i.e., 24 hours after UUO). Kidney sections were immersed in Tris-EDTA based solution, for 15 minutes at 95°C and stained with the corresponding anti-BrdU antibody (Supplemental Table 1).

Quantification of tubular cell proliferation

The tubular cell proliferation index was calculated as the number of Ki67, PCNA or BrDU positive nuclei over the total number of tubular nuclei for each nephron segment, using the "Cell Counter" plugin of ImageJ software. It was scored in at least 10 randomly selected fields per kidney section.

Quantitative tubule morphometric analysis

The tubule cross-sectional area (expressed in μm2) was automatically quantified with ImageJ2 software using U-Net Segmentation Plugin (73). Briefly, the outer tubular surface of proximal tubules (PT; brush border stained with WGA), distal convoluted tubules (DCT; stained with CALB) and collecting ducts (CD; stained with AQP2) were annotated on a few pictures of labelled kidneys. We used an Ubuntu server 18.04 with FIJI, a distribution of ImageJ, and the U-Net Segmentation plugin (https://sites.imagej.net/Falk/) that interfaces artificial neural networks. A pre-trained model (2d_cell_net_v0) was used and adjusted with the plugin option 'U-Net Finetuning' : manual

annotation on personal pictures: extpct 129, extdistal 76, glom 5. The model was applied to segment tubules in large regions of the cortex.

Quantification of internuclear distance was performed manually on each image using the Line Selection Tool in ImageJ2 software, as the distance (expressed in μm) between the centre of two adjacent nuclei along the tubular wall. It was measured for each specifically stained nephron segment of interest: the proximal tubule, the distal convoluted tubule and the collecting duct.

To facilitate the comparison of the morphometric data obtained in 8 and 12 weeks-old animals, the cross-sectional area and internuclear distance measured at 12 weeks were divided by the mean observed in control mice at 12 weeks and multiplied by the mean value observed in control mice at 8 weeks.

Measurement of TBM components staining intensity

Fluorescence intensity of TBM components (HSPG, collagen IV, laminin) was automatically quantified using a specific ImageJ2 macro, named “Do_ROI_dilate_manager”. Briefly, it consisted in surrounding the basement membranes stained with WGA of each tubule of interest on immunofluorescence kidney sections, using the Area Selection Tool in Image J2. Each selected area was extracted as a ROI (Region Of Interest), then listed in the ROI Manager and individually dilated by the macro to capture all TBM signals. For each tubule, fluorescence intensity of TBM components was assessed by the Mean Gray Value. The mean fluorescence intensity per mouse was then calculated as the mean of the mean gray values of individual tubules.

Transmission electron microscopy

Five millimeter-thick cortical slices from each kidney were fixed in 2.5% glutaraldehyde prepared in 0.1 M sodium cacodylate buffer (pH 7.4; Euromedex, 12300-25) for 24 hours at 4 °C. After rinsing with 0.1 M sodium cacodylate buffer, kidney samples were maintained in the same buffer at

4 °C. Samples were then post-fixed in 2% osmium tetroxide (OsO_4) in distilled water for 1 hour at room temperature. Following extensive washing (five times) with distilled water, samples were incubated for 1 hour in 2% uranyl acetate in distilled water. They were then dehydrated through a graded series of ethanol solutions (50%, 70%, 80%, 90%, 95%, and 100%) followed by acetone (100%), with each step lasting 10 minutes and two incubations at 100%. Samples were subsequently embedded in Epon 812® epoxy resin (EMS, Souffelweyersheim, France) as follows: overnight infiltration in a 1:1 mixture of resin and acetone at 4 °C in an airtight container, followed by two incubations of 2 hours each in pure fresh resin at room temperature. Resin blocks were sectioned using a UC7 ultramicrotome (Leica Microsystems SAS). Ultrathin sections (70 nm) were collected on copper grids, contrasted with Reynolds' lead citrate (Reynolds, 1963), and examined using a Hitachi HT7700 transmission electron microscope (Milexia, Saint-Aubin, France) operating at 100 kV. Images of proximal tubules (PT), distal convoluted tubules (DCT), and collecting ducts (CD) were acquired at a resolution of 2048 × 2048 pixels using an AMT41B camera or at 4096 × 4096 pixels using a Nanosprint12 camera, and processed with ImageJ2 software (version 2.9.0/1.53t). TEM data acquisition was performed at the Imaging Facility of Hôpital Tenon (INSERM UMRS_S1155) and at the Institut du Cerveau et de la Moelle Épinière, Sorbonne Université (INSERM U975). Mean basement membrane thickness per tubule was determined in a blinded fashion using 8 to 20 measurements per tubule. Further TEM acquisition and analysis was performed at the institute of Surgical Pathology, University Medical Center Freiburg (Hitachi HT 7800 transmission electron microscope, Japan).

Measurement of pression-diameter curves in isolated perfused tubule

Kidneys from control, *Pkd1*$^{\Delta tub}$ and *Pkd1*$^{\Delta tub}$; *Kif3a*$^{\Delta tub}$ mice were collected at 8 and 12 weeks and cut into slices. PT and CD were then dissected without collagenase digestion, to preserve TBM mechanics. Each isolated tubule was transferred to a 37°C controlled-bath chamber under an

inverted microscope (Axiovert 100, Carl Zeiss) and mounted between two concentric glass pipettes, as previously described (74). The perfusion and bath solutions were composed of 144 mM NaCl, 5.5 mM glucose, 5 mM alanine, 10 mM HEPES, 1.5 mM CaCl2, 1.2 mM MgSO4, 2 mM K2HPO4, pH 7.4. The TBM of isolated tubule was stained for 5 minutes by adding fluorescein-labelled WGA within the bath chamber (Supplemental Table 1). A solution column connected to the perfusion pipette was vertically displaced, allowing for progressive increment of transmural pressure delivered to the tubule lumen, from 0 to 42 cmH20. At each pressure point, the outer tubular diameter was measured (mean of 10 measurements per tubule).

Microdissection of tubule segments

Deeply anesthetized mice were placed under binocular microscope and submitted to a laparotomy, followed by an incision of subcutaneous and muscular layers to gain access to the peritoneal cavity. A first vascular clamp was applied to the abdominal aorta to prevent bleeding during aortic incision. Three other ligatures were placed on the abdominal aorta at different locations before aortic incision: at the distal end to facilitate microperfusion, at the mid part to prevent catheter displacement and at the proximal end before the bifurcation of the left renal artery to maximize the perfusion of the left kidney. An incision was then performed in the wall of the abdominal aorta and a catheter linked to a syringe was inserted in the lumen towards the left renal artery. The left kidney was rinsed 5 ml of dissection buffer [Hank's medium without phenol (L0612-500, Eurobio) complemented with 1 M MgCl2, 3 M sodium acetate, 100 mM sodium pyruvate, 200 mM glutamine, 1 M HEPES, 0.1% BSA, pH 7.4] and perfused with the same medium containing 120 µg/ml of Liberase (Roche, 05401127001). Thin kidney slices were cut along the corticomedullary axis and incubated at 30°C for 25 minutes in dissection buffer containing 40 µg/ml of Liberase. Kidney slices were then transferred in Petri dishes containing dissection buffer. The PTs and the CDs were microdissected

under binocular magnifier according to morphological and topographic criteria, using fine needles. Approximately 150 PTs and 150 CDs were isolated from each kidney.

RNA-seq pre-processing and analysis

Total RNAs were extracted and purified from the microdissected PTs and CDs using RNeasy Micro Kit (Qiagen; 74004) according to the manufacturer's protocol. RNA quality was verified by capillary electrophoresis using Fragment AnalyzerTM.

Bulk mRNA-Seq libraries were prepared by the Imagine Genomic Core Facility (Paris, France) starting from 7ng of total RNA, using the NEBNext® Low Input RNA Library Prep Kit according to manufacturer's guidelines. This kit generates mRNA-Seq libraries from the PolyA+fraction of the total RNA by template switch reverse transcription. The cDNA is amplified twice and the final mRNAseq libraries are not 'oriented' or 'stranded' (meaning that the information about the transcribed DNA strand is not preserved during the library preparation). The equimolar pool of libraries, assessed by Q-PCR KAPA Library Quantification kit (Roche) and with a run test using the iSeq100 (Illumina) was sequenced on a NovaSeq6000 (Illumina, S2 FlowCells, paired-end 100+100 bases, ~50 million reads/clusters produced per library). After the demultiplexing and before the mapping, the reads were trimmed to remove the adaptor sequences from the first amplification. Reads were mapped to the GRCm38 primary mouse genome assembly with STAR(75). Counts table was generated with feature Counts from subread-1.6.4 package (76). Normalization and differential gene expression (DGE) analyses were performed with R package DESeq2 v1.32.0 (doi:10.1186/s13059-014-0550-8), and Benjamini-Hochberg correction was applied with threshold for significance set at adjusted P-values < 0.05.

Based on data from bulk RNASeq generated in tubules from either *Pkd1* mutant or control mice, we identified the genes that are differentially expressed in collecting ducts and proximal tubules by using the package Deseq2 . Gene set enrichment analyses were performed on GSEA v4.3.2 software

(Broad Institute). The expression levels of the following genes were analyzed : Adamts1, Ctss, Mmp12, Mmp14, Chsy1, Cspg4, Hs3st3a1, Adamtsl4, Lama4, Lamc1, Mgp, Nid1, Ogn, Itga9, Itgal, Itgax, Itgb2, Coro1a, Coro2a, Fgd3, Fscn1, Map6, Msn, Myl9, Myo1f, Rac2, Glis2.

Analysis of BM remodeling signature expression in human single nuclei RNAseq dataset

We downloaded a single nuclei dataset from human PKD (31) on the Gene Expression Omnibus platform (GSE185948). After using a mouse-human ortholog table, we calculated a z-score of the signature for each cell from the dataset. Violin plot according to the cell type was then generated using the VlnPlot function from Seurat. We used the cell type annotations identified in the original article(31). UMAP expression plots for selected genes were generated using the KIT interactive website (http://humphreyslab.com/SingleCell/) (77).

Analysis of BM remodeling signature expression in mouse TRAP-seq dataset

We downloaded a TRAP-seq dataset from control, *Pkd1*$^{\Delta tub}$ and *Pkd1*$^{\Delta tub}$; *Kif3a*$^{\Delta tub}$ mice from the Gene Expression Omnibus platform (GSE232556)(14). Male and female mice were pooled. After normalization by the Deseq2 package we calculated a z-score of the signature described earlier according to each genotype (78). Each gene of the signature was after that plotted , and statistical analysis using a Dunn test was performed.

Tubule-on-chip fabrication and culture conditions

As previously described (33), the chip consists of a PDMS scaffold of 3 PDMS layers bonded to a glass slide. One part of the scaffold features the microfluidic channels. For this part, a PDMS (Curing Agent to PDMS weight ratio of 1:10) was poured on a mold obtained with photolithography. This part lays on a flat PDMS membrane which elevates the channels from the glass slide. This flat PDMS layer was obtained by spin coating PDMS 1:10. On top, another flat

500-µm thick PDMS membrane closes the chip. The PDMS scaffold surface was activated with oxygen plasma and then treated with a 5% (3-aminopropyl) triethoxysilane (Sigma-Aldrich) solution in methanol for 45 min, rinsed thoroughly in methanol in an ultrasonic bath then in a 2.5% glutaraldehyde (Sigma-Aldrich) solution in ddH20 for 45min and rinsed thoroughly in ddH20 in an ultrasonic bath then at room temperature overnight. Then, 75 µm wide tungsten wires were inserted into the scaffold, which was sterilized with UV-Ozone. A 6g/L or 9.5g/L collagen I gel was prepared on ice by neutralizing high concentration collagen gel from rat tail with NaOH and diluted in PBS with 10% of total volume with PBS 10X. The gel was injected in the collagen chambers, still on ice. The collagen was set at 37°C for 2 hours. Chips were then stored in PBS at 4°C up to 2 weeks before use.

The day before seeding, wires were partially removed to form the channels and a laminin 50µg/mL solution was injected into the chip using a pressure controller and PTFE tubing followed by 1 hour-incubation at 37°C. For the seeding, we used an edited clonal mIMCD-3 cell line (immortalized cells from murine inner medullary collecting duct) , deleted for *Pkd1* gene using CRISPR-based genome editing, kindly provided by Michael Köttgen (34). These cells were detached from flask upon confluency with trypsin, counted and resuspended at 10 million cells/mL in DMEM/F-12 medium with 10% FBS. The cell suspension was then injected into collagen channels with a pressure controller via PTFE tubing until the channels were filled with cells. Then tungsten wires were inserted back into the channels to form a hollow tube. Chips were incubated in DMEM/F-12 medium with 10% FBS for 2 to 3 hours to let cells adhere. Then tungsten wires were removed to obtain a confluent mIMCD-3 layer. Chips were then cultured in 6-well plates in humid atmosphere at 37°C for up to 2 weeks in DMEM/F-12 medium with 10% FBS. Medium was changed every two days. Chips were then washed with PBS twice before being fixed in a 4% paraformaldehyde solution in PBS for 45 min. Chips were then rinsed twice in PBS and stored in PBS at 4°C before immunostaining.

Tubule-on-chip immunolabelling

The cells were permeabilized by submerging the chip into PBS + 0.3% TritonX100 for 30 minutes, and rinsed three times for 30 minutes with PBS-BSA 2%. The chip was submerged in PBS-BSA 2% with 0.1% Tween20 and 2% goat serum for saturation. A 100-150 µL drop of 1/200 antibody, 2% BSA, 1% Tween20 in PBS was delicately placed on the chip so that it did not fall. Incubation was carried out for 24 hours in a humid environment at room temperature. The chip was rinsed 3 times for 30 minutes in PBS-BSA 2%. A 100 µL drop of a solution containing 1 µg/ml Hoechst, 0.25 µg/ml fluorescent phalloidin, rabbit anti-Ki67, (Abcam, ab16667, 1:100), 2% BSA, 1% Tween-20 in PBS was delicately placed to prevent spilling. Incubation was carried out for 24 hours in a humid environment at room temperature. Mouse anti-acetylated alpha-tubulin (Santa Cruz Biotechnology, sc-23950) and anti-gamma-tubulin antibodies (Sigma-Aldrich, T6557) were used to determine the percentage of ciliated cells in *Pkd1*-deficient tubules in relation to collagen content.

AI transparency statement

During revision of this manuscript, the authors used Claude (Anthropic; Claude Opus 4, model claude-opus-4-8) to assist with condensing the text to meet the journal's word limit, harmonizing gene-symbol formatting, and reorganizing methodological content into the Supplemental Methods. The tool was used under direct author supervision; it did not generate, analyze, or interpret any experimental data, and all scientific content, conclusions, and final wording were reviewed and approved by the authors, who take full responsibility for the integrity of the work.

TABLE OF CONTENTS

Supplemental Table 1. List of the antibodies and dyes used in this study

Supplemental Figure 1. Analysis of Cre expression, *Pkd1* allele recombination and cilia loss in the $Pax8^{rtTA}$;*TetO-cre*; $Pkd1^{fl/fl}$ inducible adult model

Supplemental Figure 2. Cilia ablation completely prevents $Pkd1^{-/-}$ tubule deformation at 18 weeks

Supplemental Figure 3. *Pkd1* deletion induces cilia-dependent TBM remodeling

Supplemental Figure 4. Remodeling of the tubular basement membrane (TBM) precedes tubular distension in *Pkd1*-deficient female mice

Supplemental Figure 5. RNAseq profiling of micro-dissected proximal tubules and collecting ducts

Supplemental Figure 6. *Pkd1* deletion induces overexpression of BM remodeling genes in a cilia-dependent manner

Supplemental Figure 7. RNAseq profiling of micro-dissected $Pkd1^{\Delta tub}$; $Ift20^{\Delta tub}$ proximal tubules

Supplemental Figure 8. Control and *Pkd1*-deficient tubules display an elastic behavior

Supplemental Figure 9. Acute ureteral obstruction accelerates Polycystin-1 deficient distal tubule dilation in a cilia-dependent manner in female

Supplemental Figure 10. Acute ureteral obstruction accelerates Polycystin-1 deficient distal tubule dilation in a cilia-dependent manner in male

Supplemental Figure 11. Ureteral obstruction triggers explosive cystogenesis in $Pkd1^{\Delta tub}$ distal tubules in a cilia-dependent manner

Supplemental Figure 12. Difference in cell proliferation does not explain cilia-dependent $Pkd1^{\Delta tub}$ tubule dilation after obstruction

Supplemental Table 1. List of the antibodies and dyes used in this study

Reference	Primary antibody/Dye/Lectin	Dilution	Source
sc-515770	Mouse anti-AQP2	1:200	Santa Cruz Biotechnology
sc-9882	Goat anti-AQP2	1:200	Santa Cruz Biotechnology
A7310	Rabbit anti-AQP2	1:200	Sigma-Aldrich
ab6326	Rat anti-BrdU	1:100	Abcam
sc-365360	Mouse anti-CALBINDIN	1:200	Santa Cruz Biotechnology
NB110-59981	Rabbit anti-COL4A1	1:200	Novus Biologicals
L9393	Rabbit anti-laminin	1:100	Sigma-Aldrich
H3570	Hoechst 33341	1:1000	Thermo Fisher
A kind gift of Brigitte Lelongt	Rabbit anti-HSPG	1:100	Makino H *et al* (1986)[18]
ab16667	Rabbit anti-Ki67	1:100	Abcam
M0879	Mouse anti-PCNA	1:1000	Dako
FL-1021	Fluorescein-labeled WGA	1:200	Vector Laboratories
RL-1022	Rhodamine-labeled WGA	1:200	Vector Laboratories
FL-1321	Fluorescein-labeled LTL	1:200	Vector Laboratories
orb11618	Goat anti-mCherry	1:200	Biorbyt
sc-23950	Mouse anti-acetylated α-tubulin	1:200	Santa Cruz Biotechnology
T6557	Mouse anti-ɣ-tubulin	1:200	Sigma-Aldrich

Reference	Secondary antibody	Dilution	Source
A-21202	Donkey anti-mouse, Alexa Fluor 488	1:500	Thermo Fisher Scientific
A-21447	Donkey anti-goat, Alexa Fluor 647	1:500	Thermo Fisher Scientific
A-21206	Donkey anti-rabbit, Alexa Fluor 555	1:500	Thermo Fisher Scientific
A-21208	Donkey anti-Rat, Alexa Fluor 488	1:500	Thermo Fisher Scientific
A-21240	Goat anti-Mouse IgG1, Alexa Fluor 647	1:1000	Thermo Fisher Scientific

Reference	Biotinylated antibody	Dilution	Source
RPN1004V	Biotinylated Donkey anti-rabbit	1:200	GE Healthcare

Reference	Streptavidin conjugate	Dilution	Source
S-32354	Streptavidin conjugate, Alexa Fluor 488	1:500	Thermo Fisher Scientific
S-32355	Streptavidin conjugate, Alexa Fluor 555	1:500	Thermo Fisher Scientific

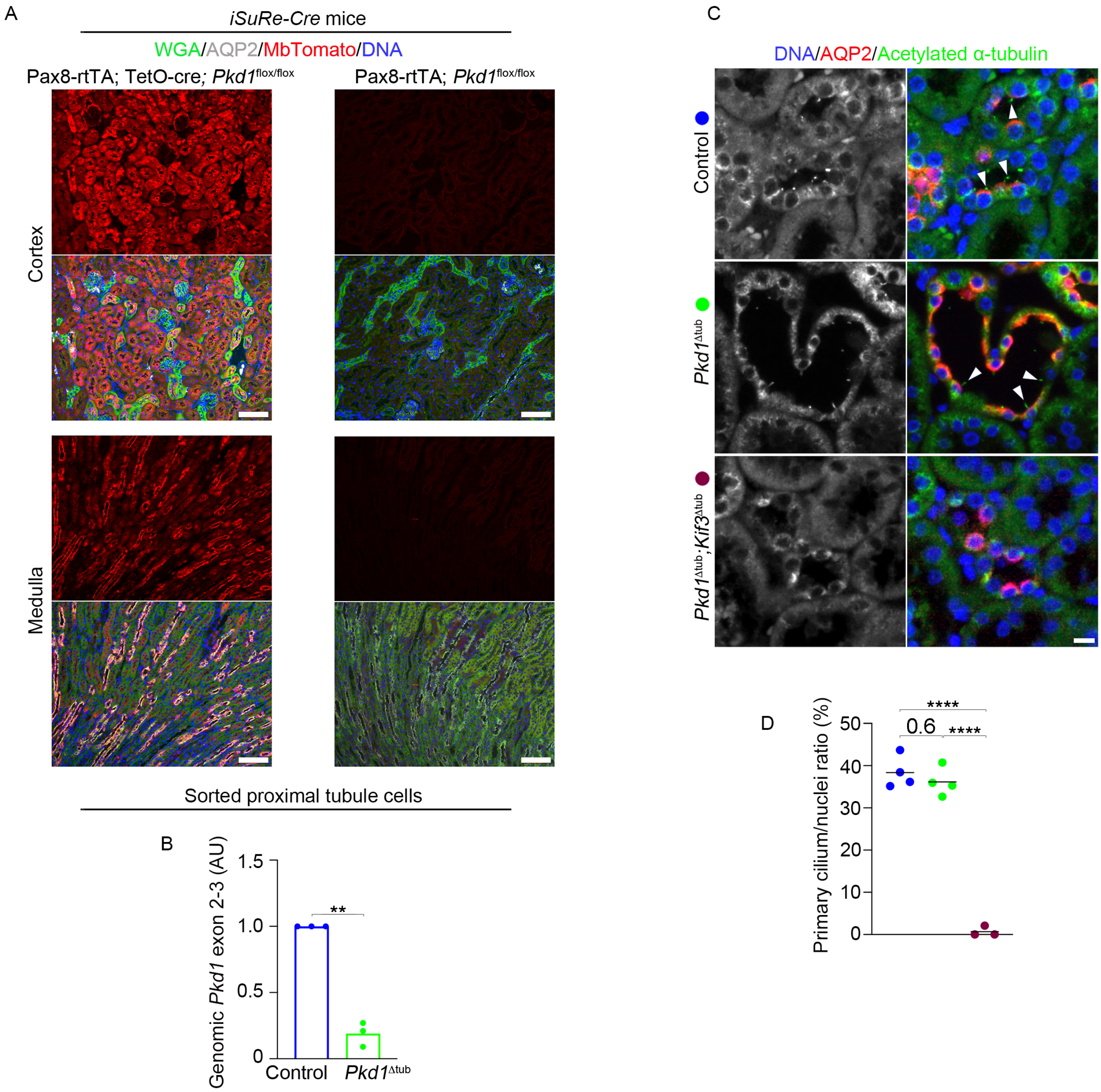

Supplemental Figure 1. Analysis of Cre activity, *Pkd1* allele recombination and cilia loss in the *Pax8^{rtTA};TetO-cre; Pkd1$^{fl/fl}$* inducible adult model. (**A**) Labelling of membrane Tomato (MbTomato; whose expression is induced by the *iSure-Cre* reporter upon cre-mediated recombination), wheat germ agglutinin (WGA, which stains the proximal tubule [PT] brush border and all basement membranes), and aquaporin-2 (AQP2, a collecting duct [CD] marker) in kidneys from *Pkd1*$^{\Delta tub}$; *iSure-Cre* mice or control *iSure-Cre* mice lacking the TetO-Cre transgene, two weeks following the end of doxycycline treatment. Scale bar: 50 µm. (**B**) qPCR quantification *Pkd1* exon 2-3 excision on genomic DNA from proximal tubular cells sorted from the kidneys of 6-weeks-old control and *Pkd1*$^{\Delta tub}$ mice. Each dot represents one mouse. Student t-test : **P<0.01(**C** and **D**). Labelling of primary cilia (acetylated-α-tubulin), AQP2 and DNA and quantification of primary cilia to nuclei ratio in AQP2+ tubule sections from 8-week-old control, *Pkd1*$^{\Delta tub}$ and *Pkd1*$^{\Delta tub}$; *Kif3a*$^{\Delta tub}$ mice. Each dot represents one mouse. Scale bar: 10 µm. One-way ANOVA followed by Tukey-Kramer test: ****P<0.0001 or the indicated P-value.

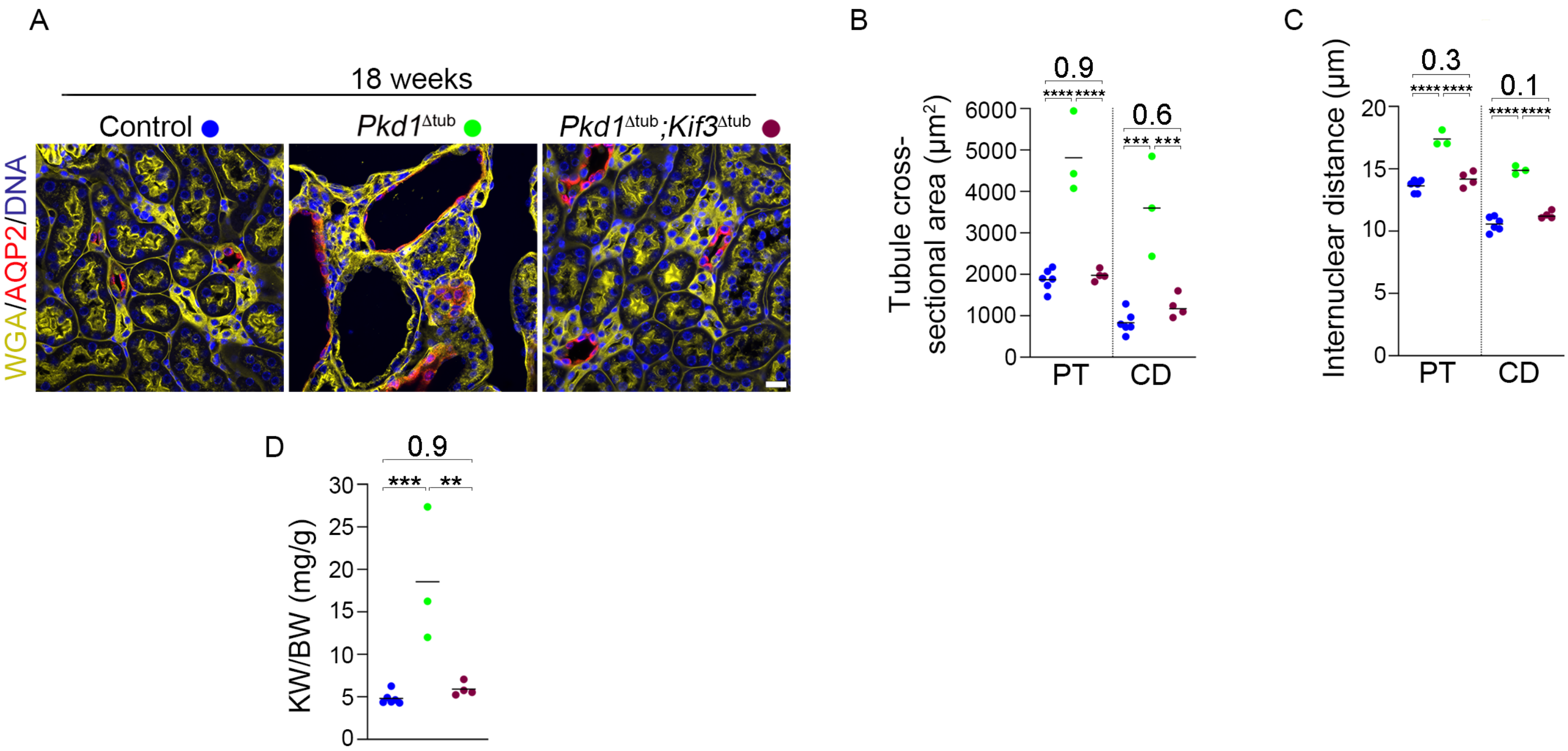

Supplemental Figure 2. Cilia ablation completely prevents *Pkd1*$^{-/-}$ tubule deformation at 18 weeks. (**A**) Labelling of DNA, wheat germ agglutinin (WGA, which stains the brush border of proximal tubule [PT] and all basement membranes) and aquaporin 2 (AQP2, a collecting duct [CD] marker) of kidneys from 18-week-old control, *Pkd1*$^{\Delta tub}$ and *Pkd1*$^{\Delta tub}$; *Kif3a*$^{\Delta tub}$. Scale bar: 20 µm. (**B** and **C**) Quantification of mean PT and CD cross-sectional area (B) and internuclear distance (C) in kidneys from the same animals. (**D**) Quantification of KW/BW ratio of kidneys from the same animals. Each dot represents one individual male mouse. One-way ANOVA followed by Tukey-Kramer test: **P<0.01, ***P<0.001, ****P<0.0001 or the indicated P-value. Control mice consist in *Pkd1*$^{flox/flox}$and *Pkd1*$^{flox/flox}$;*Kif3a*$^{flox/flox}$ littermates lacking cre or rtTA transgene.

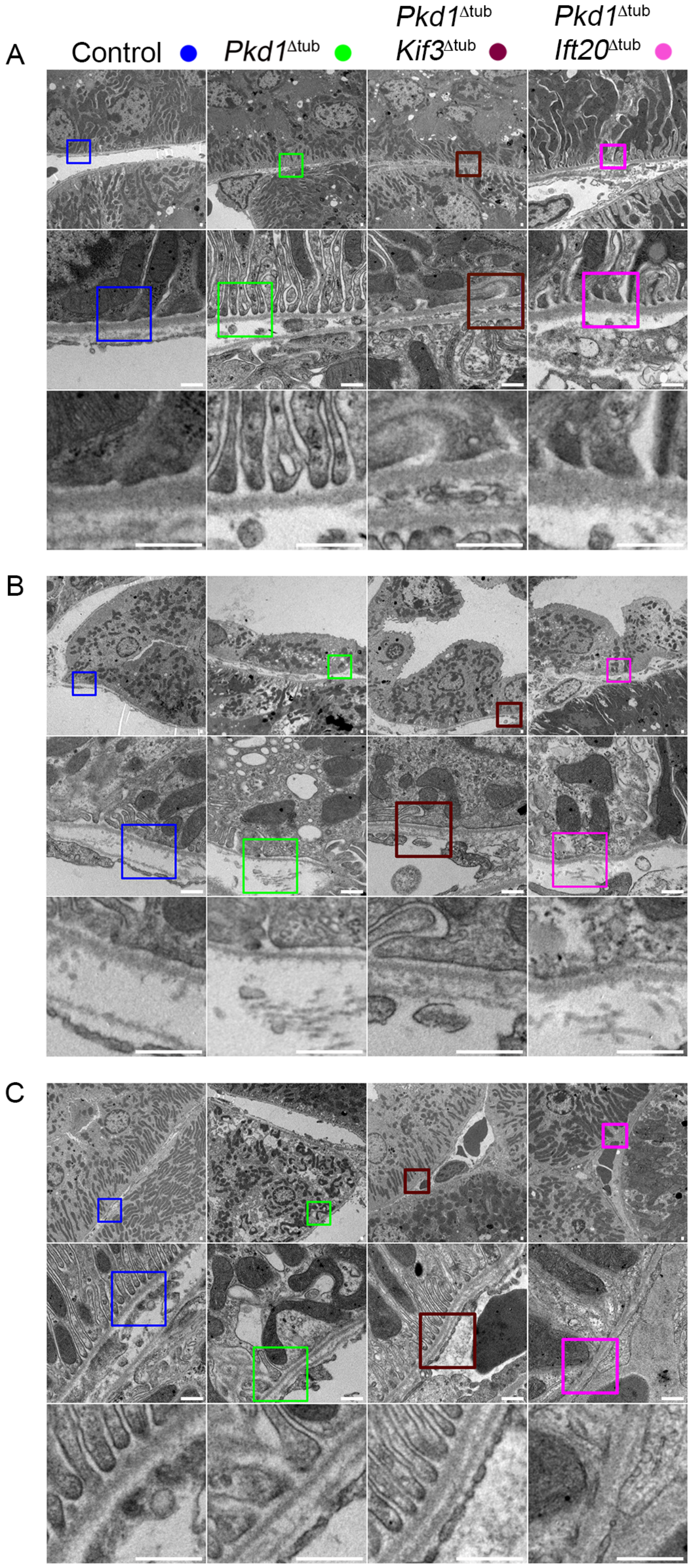

Supplemental Figure 3. *Pkd1* deletion induces cilia-dependent TBM thinning. Transmission electron microscopy of basement membrane thickness in proximal tubule (**A**), collecting duct (**B**) and distal convoluted tubule (**C**) from 8-week-old control, *Pkd1*$^{\Delta tub}$, *Pkd1*$^{\Delta tub}$; *Kif3a*$^{\Delta tub}$ and *Pkd1*$^{\Delta tub}$; *Ift20*$^{\Delta tub}$ mice. Scale bar: 0.5 µm. Control mouse consist in *Pkd1*$^{flox/flox}$ littermates lacking cre or rtTA transgene. This figure includes data previously presented in Figure 2.

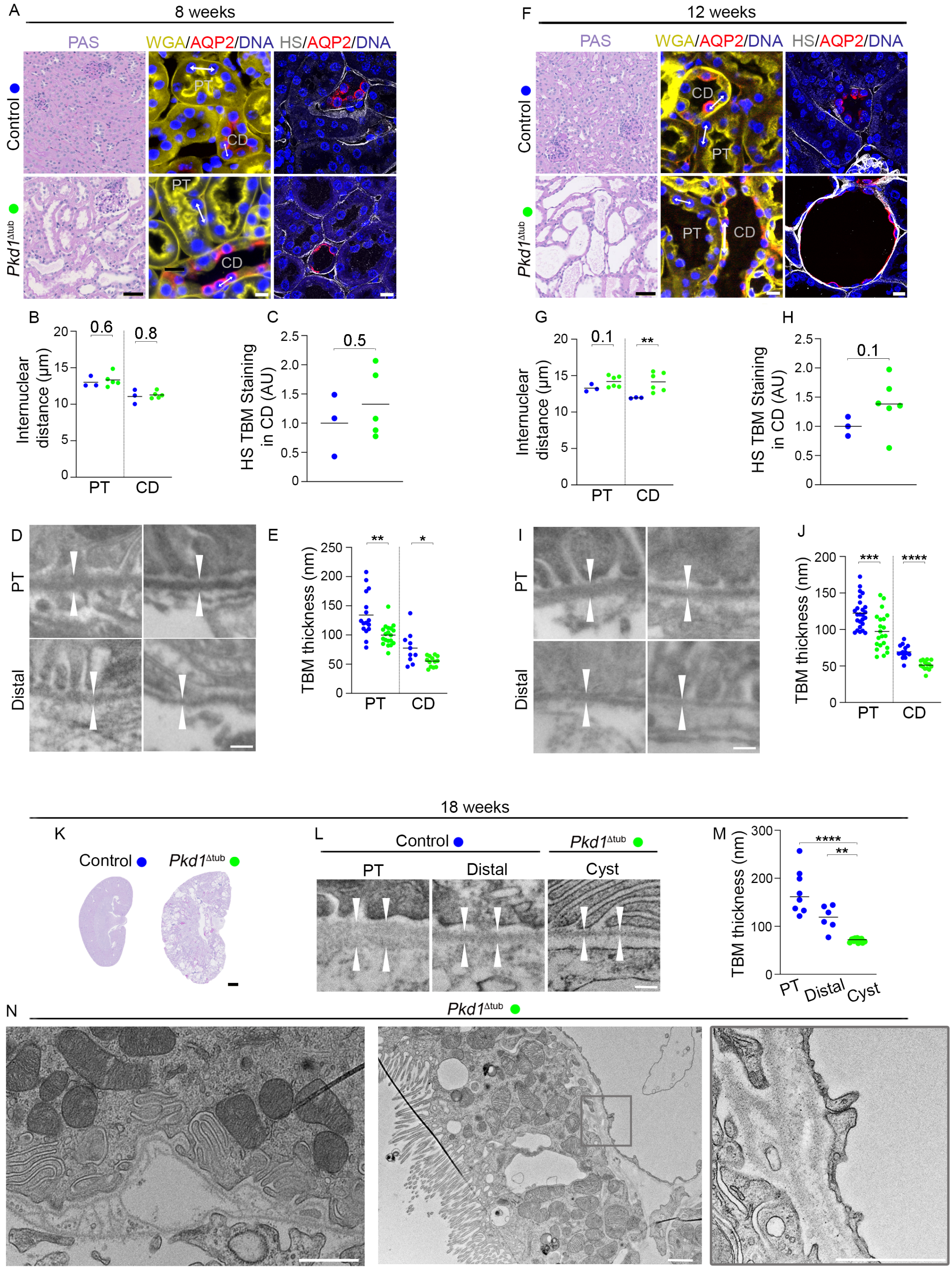

Supplemental Figure 4. Remodeling of the tubular basement membrane (TBM) precedes tubular distension in *Pkd1*-deficient female mice. (**A**) Periodic acid Schiff's (PAS) staining and labelling of DNA, wheat germ agglutinin (WGA, which stains the brush border of proximal tubule [PT] and all basement membranes), aquaporin 2 (AQP2, a collecting duct [CD] marker) DNA and heparan sulfate (HS) of kidneys from 8-week-old control and *Pkd1*$^{\Delta tub}$ female mice. Scale bars: 50 µm (PAS) and 10 µm (immunofluorescence). (**B** and **C**) Quantification of internuclear distance (B) and HS staining in collecting ducts (C) in kidneys from the same groups of mice. Each dot represents one individual mouse. Indicated P values were derived from a Student t-test. (**D** and **E**) Transmission electron microscopy (D) and quantification of TBM thickness (E) in PT and distal tubules in kidneys from the same groups of mice. Scale bar: 200 nm. Student t-test : *P<0.05, **P<0.01 or the indicated P-value. (**F**) PAS staining, labelling of PT, CD, DNA and HS in collecting ducts of kidneys from 12-week-old control and *Pkd1*$^{\Delta tub}$ female mice. Scale bars: 50 µm (PAS) and 10 µm (immunofluorescence). (**G** and **H**) Quantification of internuclear distance (G) and HS staining in collecting ducts (H) in kidneys from the same groups of mice. Each dot represents one individual mouse. Student t-test : **P<0.01. (**I** and **J**) Transmission electron microscopy (I) and quantification of TBM thickness (J) in PT and distal tubules in kidneys from the same groups of mice. Scale bar: 200 nm. Student t-test : *** P<0.001, ****P<0.0001. (**K**) PAS staining of kidneys from 18-week-old control and *Pkd1*$^{\Delta tub}$ female mice. Scale bar: 1 mm. (**L** and **M**) Transmission electron microscopy (L) and quantification of TBM thickness (M) in PT, distal and cystic tubules in kidneys from the same groups of mice. In TBM thickness graphs, each dot represents one individual tubule (mean of 8 to 20 measurements per tubule; n=3 mice per genotype). Scale bar: 200 nm. One-way ANOVA followed by Tukey-Kramer test: **P<0.01, ****P<0.0001. Control mice consist in *Pkd1*$^{flox/flox}$ littermates lacking cre or rtTA transgene. (**N**) Representative transmission electronic microscopy images showing complex TBM remodeling in kidney cyst from 18 weeks *Pkd1*$^{\Delta tub}$ female mice. Scale bars: 1 µm.

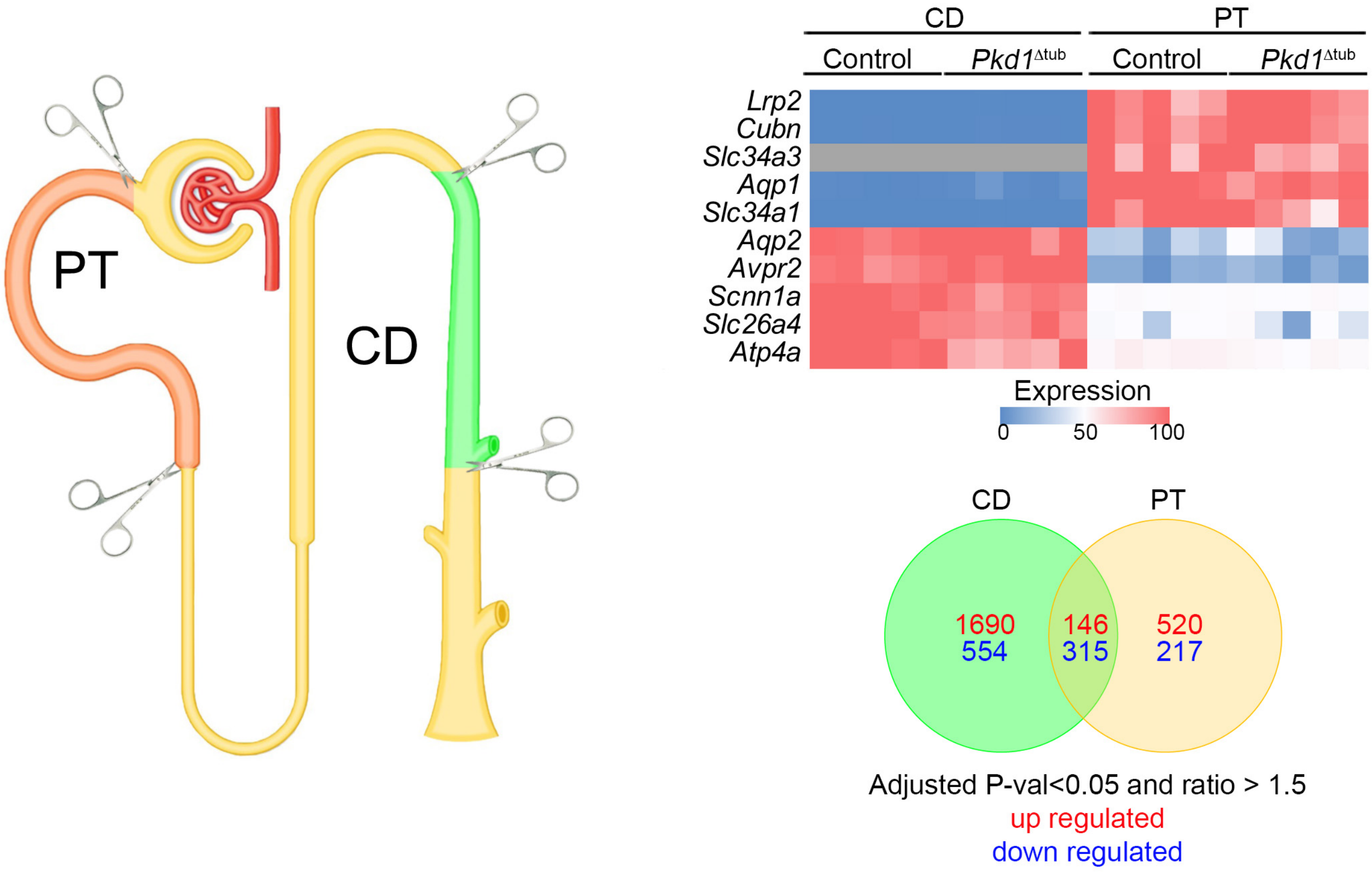

Supplemental Figure 5. RNAseq profiling of micro-dissected proximal tubules and collecting ducts. (**A**) Schematic representation of the dissected segments. (**B**) Heatmap of the relative expression of proximal tubules (PT) and collecting ducts (CD) specific genes in micro-dissected tubules. (**C**) Venn diagram showing the number of genes with significant variation between *Pkd1* mutant animals and controls in CD and PT. Control mice consist in *Pkd1*$^{flox/flox}$ littermates lacking cre or rtTA transgene.

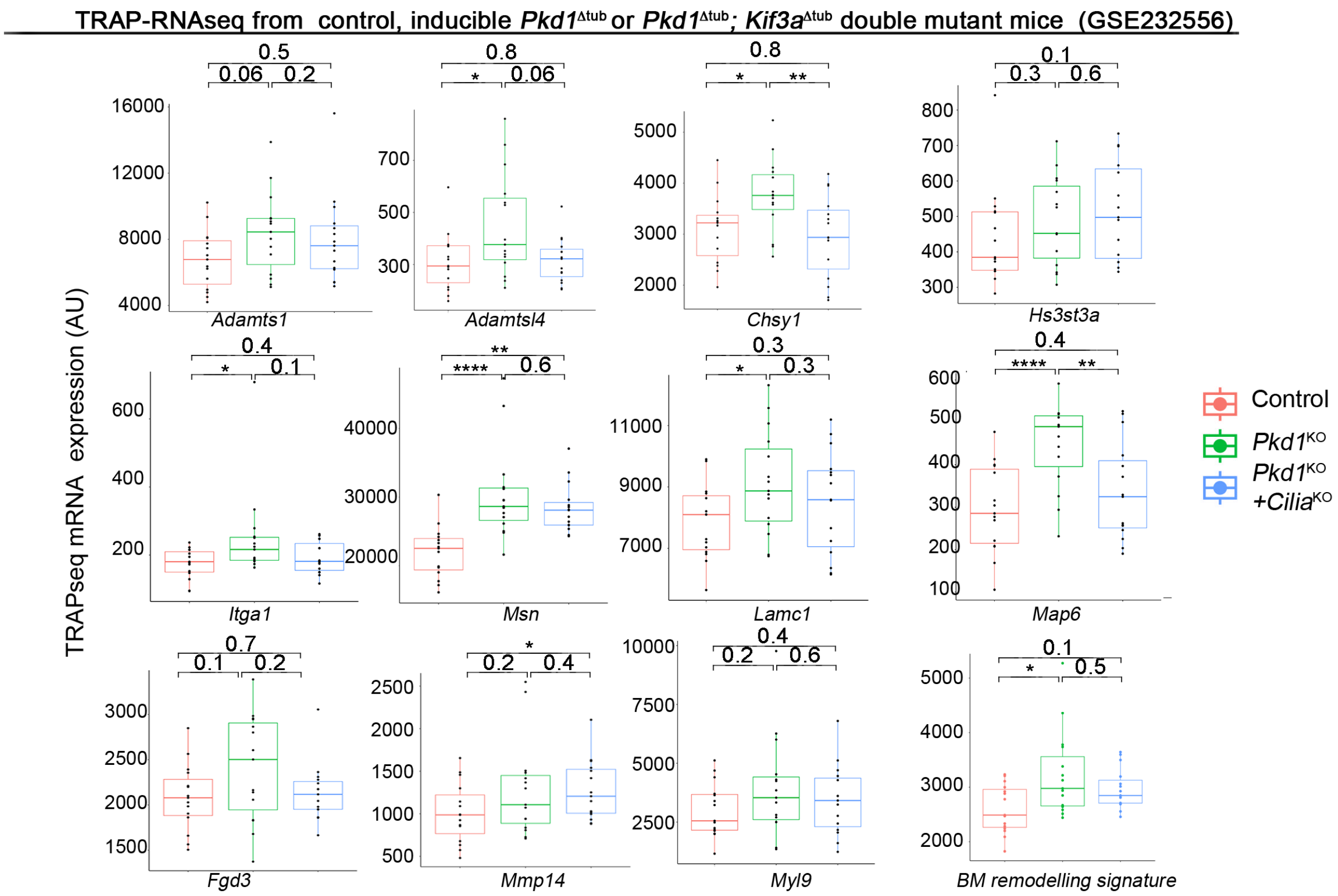

Supplemental Figure 6. *Pkd1* deletion induces overexpression of BM remodeling genes in a cilia-dependent manner. Re-analysis of translating ribosome affinity purification (TRAP-Seq) of kidney tubule translatome of control, *Pkd1*$^{\Delta tub}$ and *Pkd1*$^{\Delta tub}$; *Kif3a*$^{\Delta tub}$ 10-week-old male mice showing the expression of selected genes associated with BM remodeling.

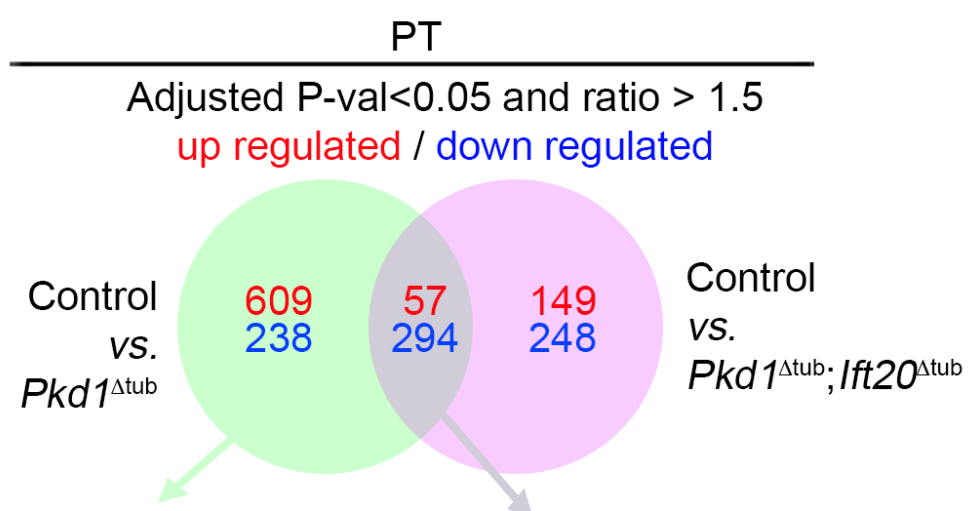

Cilia dependent

Mxra7, Ctxn3, Egfl6, Grasp, Icosl, Mmp14, Uhrf1, Npas1, Dbf4, Scin, Shh, Plin4, Pla1a, Slc2a3, Dusp3, Fosb, Etv1, Hif3a, Cnn2, Matk, Klf5, Slc1a3, Il5ra, Asf1b, Cyp2a5, Runx1t1, Cyp27b1, Hap1, Pole, Cpt1c, Rarres2, Spaca7, Odc1, Kif11, Igf2bp1, Glis2, Stc1, Map2, 1700016C15Rik, Brca1, Cdc6, Mybl2, Ptgis, Kcnab3, Dnah11, Ikzf1, Scrn1, Chtf18, Igf1, Prss57, Adcy1, Osbp2, Prr11, Xylt2, Usp43, Cdkl1, 4930512B01Rik, Rdh12, Slc10a1, Cdca7l, Akr1c12, Akr1c13, Rgs6, Papln, Ogn, Wrnip1, Fam217a, Trip13, Tert, Hapln1, Plk2, Thbs4, Map3k1, Samd4, Ska3, Enox1, Diaph3, Esco2, Gpc5, Scel, Shcbp1, Wnt7b, Gtse1, Slc38a4, Ly6f, Psca, Arc, Serpind1, Fstl1, Chaf1b, Racgap1, Cdca3, Cldn6, Pkmyt1, Sgo1, Cyp39a1, Calcr, Cpne5, Abcg1, Ndc80, Lbh, Strn, Abcg8, Cyp4f14, Ltb, Jakmip2, Dtwd2, Slc14a2, Aldh1a7, Cdca5, Kif20b, Cabp2, Hells, Padi1, Ifitm1, Arhgef9, Alox5, Ccr1, Homer2, Sgo2a, Bard1, Iqca, Tmem163, Ube2t, Pik3c2b, Cdc42bpa, Mpzl1, Nek2, Irf6, Mrc1, 1700007K13Rik, Frzb, Jag1, Nusap1, Knl1, Fbln7, Gins1, Cdk5rap1, Aurka, Impa1, Fam83d, Zmat3, Slc6a17, Tlr2, Iqgap3, Fmo5, Tspan5, Trp53inp1, Epha7, Smc2, Atp8b5, Glipr2, B4galt2, Cdkn2c, Pdpn, Kif2c, Cyp4b1, Sema3c, Cdca8, Padi2, Slc25a33, Angptl7, Mxra8, Bst1, Afap1, Htra3, Pcdh7, Stk32b, Cenpa, Pi4k2b, Hsd17b11, Dck, Cxcl1, P2rx7, Lfng, Tspan12, Eln, Cyp3a13, Akr1b8, Aoc1, Emp1, Slc27a5, Ddias, Kif22, Lat, Dkk3, Plk1, Ubfd1, Rrm1, D7Ertd443e, Mki67, Dock11, Itm2a, Cenpi, Col4a6, Zfp185, Dusp4, Scrg1, Hpgd, Mylk3, Gpt2, 6430548M08Rik, Map3k21, Panx1, Oaf, St3gal4, Chek1, Rab27a, Pstpip1, Rab6b, Colgalt2, Chpf, Cip2a, Angptl1, Ndn, Nat8f2, Aspm, Tpst1, Lrrc58, Igsf9b, Trim59, Gbp8, Eda2r, Pkd2, Map4k5, BC030867, Ccl5, Dcbld2, Fam124a, Gnptab, Ccl2, Fignl1, Slc25a21, Aldh1b1, Muc20, Melk, Krt20, Mgat5, Ska1, Wtip, Micall2, Anln, Dchs1, Rap2b, Map11, Frem2, Azgp1, Myom3, Spry1, Galnt6, Tacc3, Tedc1, Tbx10, Ppp1r16b, Wnk2, Ptpdc1, Hsd3b5, Ttk, Egr1, Sct, Ephx1, Prc1, Cables2, Sptlc3, Stx11, Sectm1b, Mreg, Neil3, Ncam1, Msantd3, Timeless, Ptger3, Bub1b, Pclaf, Kazn, St8sia4, Slc7a13, Nbl1, Chrnb1, Arhgap11a, Slc7a1, Mdfic, L3mbtl4, Pgm5, Phlda3, Oasl1, Mcm3, Rapgef5, Ncapg2, Ctf1, Clspn, D930007J09Rik, Aqp6, Rhbdl2, Rbm20, Col6a6, Bmerb1, Proca1, 4933427E11Rik, Csrnp2, Arhgef37, Proser2, Cenpe, Mis18bp1, Nccrp1, Catsper4, Rgmb, Ckap2l, Pou4f1, Omd, Fam171b, Cdca2, Apol10b, Cited1, Ercc6l, Msrb3, Zgrf1, Cox6b2, Sema5b, Dnah3, Aldh1a1, Phgdh, Tll1, D630008O14Rik, Tceal5, Rdh19, Fabp1, Akr1c20, Map6, Zcchc24, Cdca7, Lig1, Ces1g, Bcl2, Uxs1, Lepr, C1qtnf3, Nkapl, Tmprss9, Ctf2, Serpina6, Cdh3, Ifit3b, Nsl1, Cnr2, Ncs1, Shisal1, Ces2f, Phactr2, Cped1, Mro, Tnnt1, Ifitm7, Frat1, Tspan6, Ankrd23, Cenpm, Myadm, Psrc1, Slfn9, Nlrp1a, Ccnd1, Dnajc25, Hrct1, Serpina1b, Klrg2, 6720489N17Rik, Ccnf, Ang, Ppp1r3e, Zfp951, Oip5, Lrrc61, 2500004C02Rik, C4b, Mxra8os, Cenps, Cdkn2b, Zfp382, Tmem267, Lrrc31, Tspyl3, Slc4a11, Gas2l3, Fibin, Cenpw, Prkcg, Tgtp1, Cntf, Kifc1, Mipepos, Rptoros, Smc2os, Platr21, Kcnab3os, 4930486I03Rik, A730090N16Rik, Kcne1l, 1700071M16Rik, A930033H14Rik, Gcnt4, Tmem207, Mir5620, 4931403E22Rik, 9430069I07Rik, 1810049J17Rik, D630011A20Rik, C430014B12Rik, 5430400D12Rik, 9530036O11Rik, Lockd, Mir7020, 1700054A03Rik, 4932411K12Rik, 4930444A19Rik, Thap6, Naaladl2, 5033403H07Rik, 1700126G02Rik, D030045P18Rik, Eprn

Cilia independent

Spag5, Ccne1, Cd44, Mcm5, Cdc20, Gprc5b, Ptgds, Birc5, Cdk1, Ptprr, Hmmr, Btg2, Rrm2, Aurkb, Top2a, Fos, Cxcl14, Pbk, Aqp4, Ptpn14, Cenpf, Atf3, Nuf2, Vim, Knstrn, Tpx2, Ect2, Vcam1, Car9, Hsd17b14, Ccnb2, Kif23, Ncaph, Dtl, Dlgap5, Cdkn3, Ckap2, Hspb2, Rccd1, Ccnb1, Adora1, Ifit2, Socs3, Gpc3, Nrm, 5730437C11Rik

Supplemental Figure 7. RNAseq profiling of micro-dissected *Pkd1*$^{\Delta tub}$; *Ift20*$^{\Delta tub}$ proximal tubules. Venn diagram showing the number of genes with significant variation between *Pkd1* or *Pkd1*;*Ift20* mutant animals and their corresponding littermate controls in proximal tubules (PT; upper panel). The cilia-dependent and cilia-independent genes of the basement membrane remodeling signature are indicated in the lower panel. Control mice consist in *Pkd1*$^{flox/flox}$ littermates lacking cre or rtTA transgene.

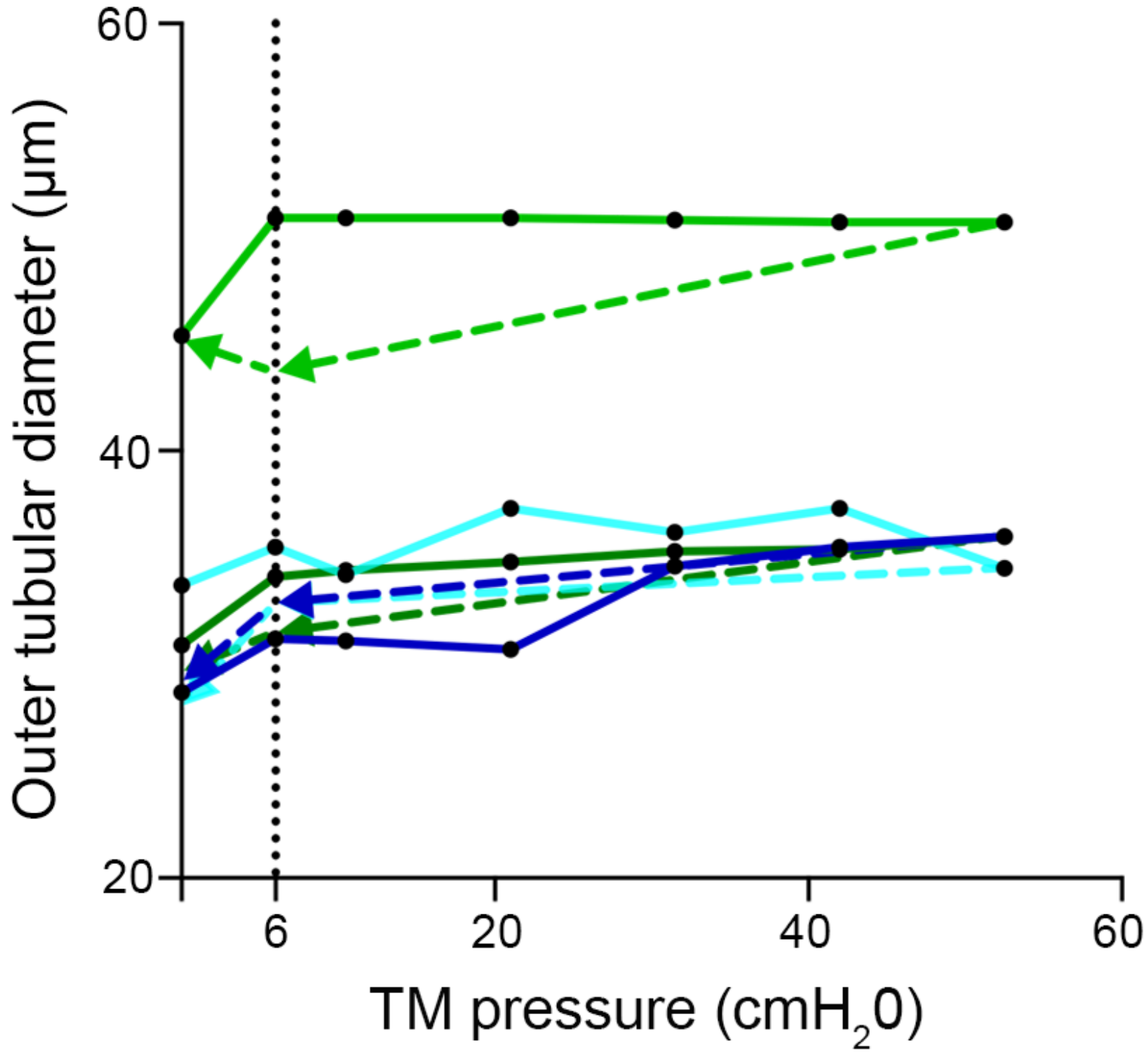

Supplemental Figure 8. Control and *Pkd1*-deficient tubules display an elastic behavior. Once luminal pressure has been increased (solid lines), releasing the pressure (dotted line) reduces tubules diameters. The diameter-transmural (TM) pressure curves of isolated collecting ducts from 8-week-old control (n=2; blue) and *Pkd1*$^{\Delta tub}$ (n=2; green) mice are represented. Control mice consist in *Pkd1*$^{flox/flox}$ littermates lacking cre or rtTA transgene.

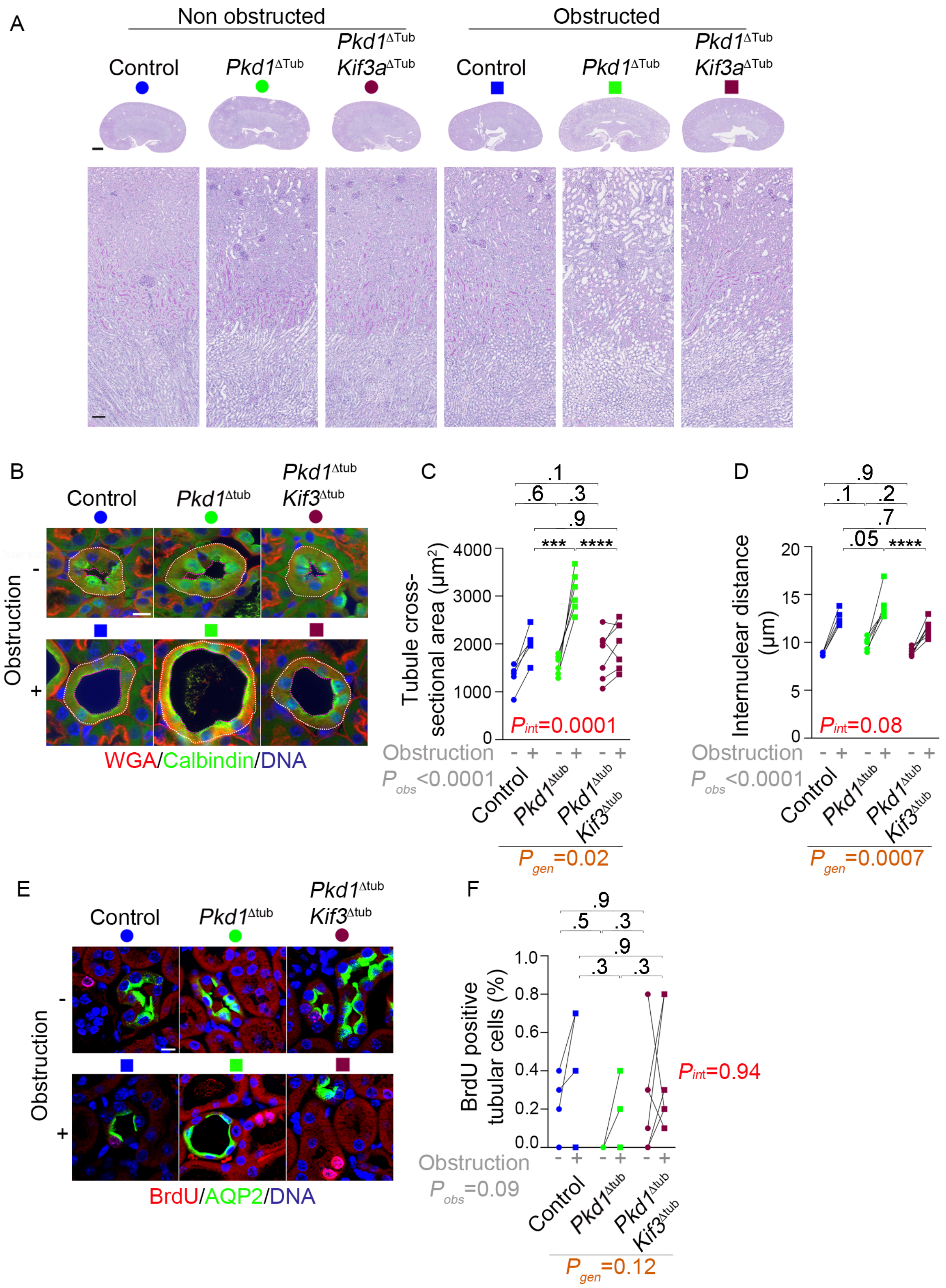

Supplemental Figure 9. Acute ureteral obstruction accelerates Polycystin-1 deficient distal tubule dilation in a cilia-dependent manner in female. (**A**) Periodic-acid Schiff's (PAS) staining of obstructed and non-obstructed kidneys from 8-week-old control, $Pkd1^{\Delta tub}$ and $Pkd1^{\Delta tub}$; $Kif3a^{\Delta tub}$ one day after unilateral ureteral obstruction (UUO). Scale bars: 1 mm (up) and 0.1 mm (down). (**B**) Labelling of DNA, wheat germ agglutinin (WGA, which stains the brush border of proximal tubule [PT] and all basement membranes) and calbindin (a distal convoluted tubule [DCT] marker) of obstructed and non-obstructed kidneys from 8-week-old control, $Pkd1^{\Delta tub}$ and $Pkd1^{\Delta tub}$; $Kif3a^{\Delta tub}$ mice one day after unilateral ureteral obstruction (UUO). Scale bar: 10 µm. (**C** and **D**) Quantification of the mean tubule cross-sectional area (D) and the internuclear distance in DCT (D) of kidneys from the same groups of animals. (**E** and **F**) Labelling of bromodeoxyuridine (BrdU), DNA and aquaporin2 (AQP2; a collecting duct marker; E) and quantification (F) of BrdU staining in collecting ducts of obstructed and non-obstructed kidneys from 8-week-old control, $Pkd1^{\Delta tub}$ and $Pkd1^{\Delta tub}$; $Kif3a^{\Delta tub}$ mice one day after unilateral ureteral obstruction (UUO). Scale bar: 10 µm. Each pair of linked symbols (dot and square) represents the obstructed (square) and non-obstructed (dot) kidneys of an individual female mouse. Two-way ANOVA, P value for obstruction (grey; P_{obs}), genotype (brown; P_{gen}) and their interaction (red; P_{int}), followed by Tukey-Kramer test: *P<0.05, **P<0.01, ***P<0.001, ****P<0.0001 or the indicated P-value. Control mice consist in $Pkd1^{flox/flox}$ and $Pkd1^{flox/flox}$;$Kif3a^{flox/flox}$ littermates lacking cre or rtTA transgene.

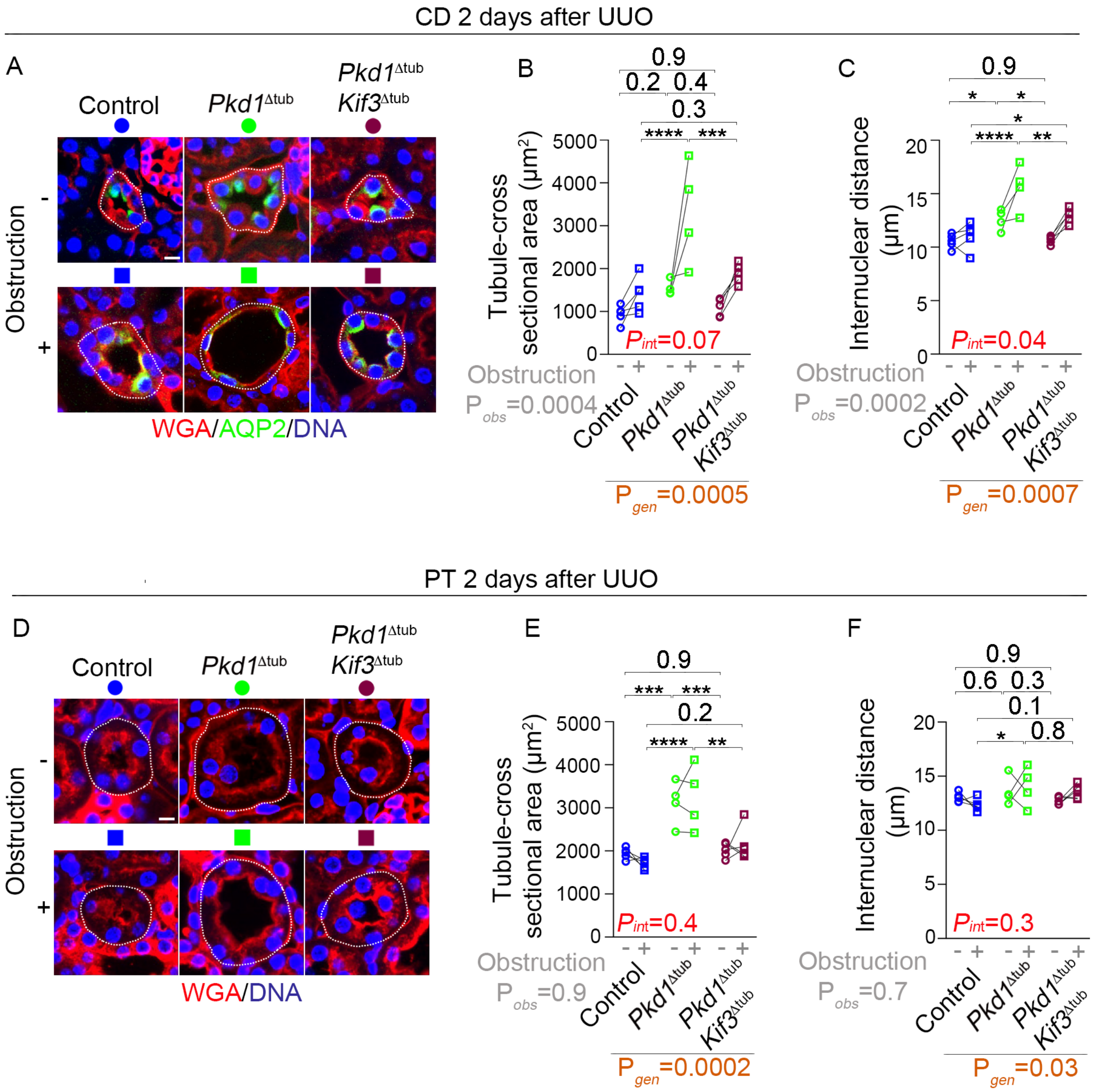

Supplemental Figure 10. **Acute ureteral obstruction accelerates Polycystin-1 deficient distal tubule dilation in a cilia-dependent manner in male.** (**A-C**) Labelling of DNA, wheat germ agglutinin (WGA, which stains the brush border of proximal tubule [PT] and all basement membranes) and aquaporin 2 (AQP2, a collecting duct [CD] marker; A), quantification of mean tubule cross-sectional area (B) and internuclear distance of CD (C) in kidneys from 8-week-old control, *Pkd1*$^{\Delta tub}$ and *Pkd1*$^{\Delta tub}$; *Kif3a*$^{\Delta tub}$ two days after unilateral ureteral obstruction (UUO). (**D-F**) Labelling of DNA and WGA (D), quantification of mean tubule cross-sectional area (E) and internuclear distance of PT (F) in kidneys from the same animals. Scale bar: 10 µm. Each pair of linked symbols (dot and square) represents the obstructed (square) and non-obstructed (dot) kidneys of an individual male mouse. Two-way ANOVA, P-value for obstruction (grey; P_{obs}), genotype (brown; P_{gen}) and their interaction (red; P_{int}), followed by Tukey-Kramer test: *P<0.05, **P<0.01, ***P<0.001, ****P<0.0001, or the indicated P-value. Control mice consist in *Pkd1*$^{flox/flox}$and *Pkd1*$^{flox/flox}$;*Kif3a*$^{flox/flox}$ littermates lacking cre or rtTA transgene.

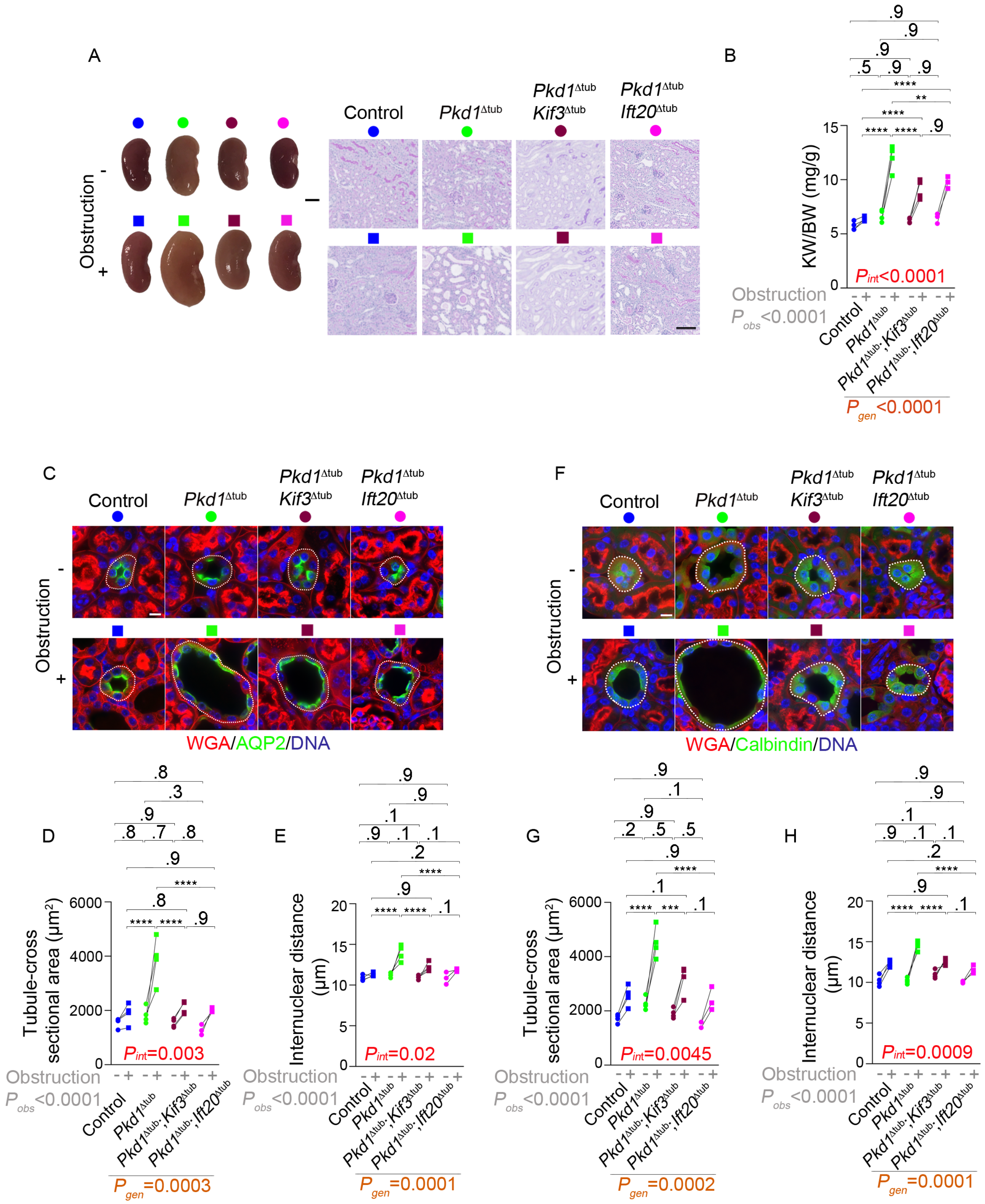

Supplemental Figure 11. Ureteral obstruction triggers explosive cystogenesis in *Pkd1*$^{\Delta tub}$ distal tubules in a cilia-dependent manner. (**A** and **B**) Kidney pictures and Periodic-acid Schiff's staining (A) and quantification (B) of kidney weight to body weight ratio (KW/BW) of obstructed and non-obstructed kidneys from 8 weeks-old control, *Pkd1*$^{\Delta tub}$, *Pkd1*$^{\Delta tub}$; *Kif3a*$^{\Delta tub}$ and *Pkd1*$^{\Delta tub}$; *Ift20*$^{\Delta tub}$ mice four days after unilateral ureteral obstruction (UUO). Scale bars: 1 mm (left) and 0.1 mm (right). Each dot represents one individual female mouse. (**C-H**) Labelling of DNA, wheat germ agglutinin (WGA, which stains the brush border of proximal tubule [PT] and all basement membranes), aquaporin 2 (AQP2, a collecting duct [CD] marker; C) or calbindin (a distal convoluted tubule [DCT] marker; F) and quantification of the mean tubule cross-sectional area (D, G) and internuclear distance (E, H) of CD (D-E) and DCT (G, H) in obstructed and non-obstructed kidneys from control, *Pkd1*$^{\Delta tub}$, *Pkd1*$^{\Delta tub}$; *Kif3a*$^{\Delta tub}$ and *Pkd1*$^{\Delta tub}$; *Ift20*$^{\Delta tub}$ mice four days after UUO. Each pair of linked symbols (dot and square) represents the obstructed (square) and non-obstructed (dot) kidneys of an individual female mouse. Scale bars: 10 µm. Two-way ANOVA, P value for obstruction (grey; P_{obs}), genotype (brown; P_{gen}) and their interaction (red; P_{int}), followed by Tukey-Kramer test: *P<0.05, **P<0.01, ***P<0.001, ****P<0.0001 or the indicated P-value. Control mice consist in *Pkd1*$^{flox/flox}$ and *Pkd1*$^{flox/flox}$;*Kif3a*$^{flox/flox}$ littermates lacking cre or rtTA transgene. This figure includes data previously presented in Figures 11 and 12.

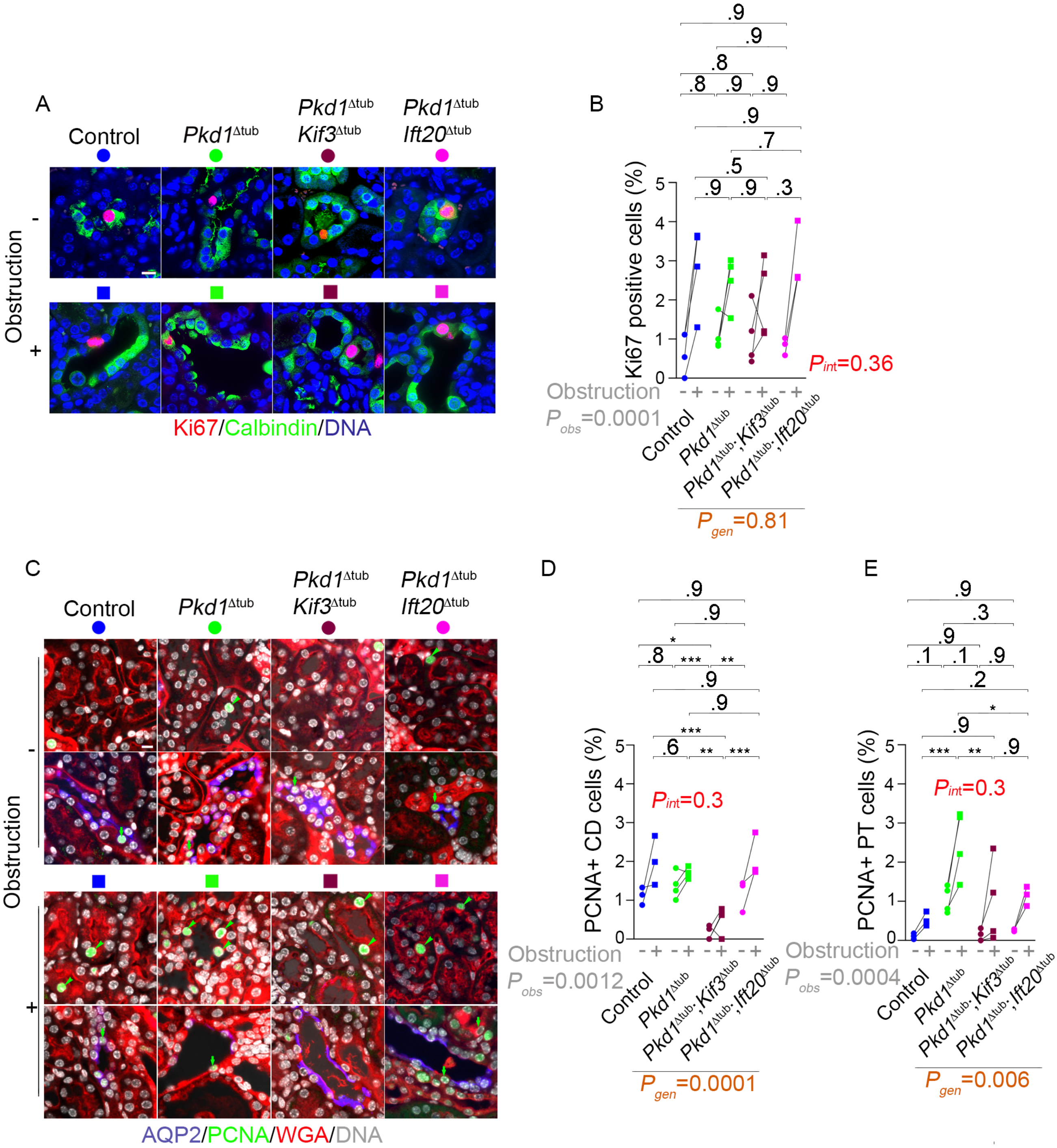

Supplemental Figure 12. Difference in cell proliferation does not explain cilia-dependent *Pkd1*$^{\Delta tub}$ tubule dilation after obstruction. (**A** and **B**) Labelling (A) of Ki67, DNA, and calbindin (a distal convoluted tubule [DCT] marker) and quantification (B) of Ki67 staining in DCT of obstructed and non-obstructed kidneys from control, *Pkd1*$^{\Delta tub}$, *Pkd1*$^{\Delta tub}$; *Kif3a*$^{\Delta tub}$ and *Pkd1*$^{\Delta tub}$; *Ift20*$^{\Delta tub}$ mice, four days after unilateral ureteral obstruction performed at 8 weeks of age. (**C-E**) Representative labelling (C) of proliferation cell nuclear antigen (PCNA), DNA, wheat germ agglutinin (WGA, which stains the brush border of proximal tubule [PT] and all basement membranes) and aquaporin 2 (AQP2, a collecting duct [CD] marker) and quantification of PCNA staining in CD (D; green arrows) and PT (E; green arrowheads) of obstructed and non-obstructed kidneys from the same animals. Each pair of linked symbols (dot and square) represents the obstructed (square) and non-obstructed (dot) kidneys of an individual female mouse. Scale bars: 10 µm. Two-way ANOVA, P value for obstruction (grey; P_{obs}), genotype (brown; P_{gen}) and their interaction (red; P_{int}), followed by Tukey-Kramer test: *P<0.05, **P<0.01, ***P<0.001, ****P<0.0001 or the indicated P-value. Control mice consist in *Pkd1*$^{flox/flox}$ and *Pkd1*$^{flox/flox}$;*Kif3a*$^{flox/flox}$ littermates lacking cre or rtTA transgene.
